# Integrative HPLC profiling and transcriptome analysis revealed insights into anthocyanin accumulation and key genes at three developmental stages of black rice (*Oryza sativa*. L) caryopsis

**DOI:** 10.3389/fpls.2023.1211326

**Published:** 2023-08-30

**Authors:** Enerand Mackon, Guibeline Charlie Jeazet Dongho Epse Mackon, Yuhang Yao, Yongqiang Guo, Yafei Ma, Xianggui Dai, Tahir Hussain Jandan, Piqing Liu

**Affiliations:** ^1^ State Key Laboratory of Conservation and Utilization of Subtropical Agro-Bioresources, College of Life Science and Technology, Guangxi University, Nanning, China; ^2^ State Key Laboratory of Conservation and Utilization of Subtropical Agro-Bioresources, College of Agriculture, Guangxi University, Nanning, China

**Keywords:** black rice, anthocyanin profiling, secondary metabolites, transcriptome analysis, HPLC, anthocyanin biosynthesis genes

## Abstract

**Introduction:**

Anthocyanins are plants' secondary metabolites belonging to the flavonoid class with potential health-promoting properties. They are greatly employed in the food industry as natural alternative food colorants for dairy and ready-to-eat desserts and pH indicators. These tremendous advantages make them economically important with increasing market trends. Black rice is a rich source of anthocyanin that can be used to ensure food and nutritional security around the world. However, research on anthocyanin accumulation and gene expression during rice caryopsis development is lacking.

**Methods:**

In this study, we combined high-performance liquid chromatography (HPLC) and transcriptome analysis to profile the changes in anthocyanin content and gene expression dynamics at three developmental stages (milky, doughy, and mature).

**Results:**

Our results showed that anthocyanin accumulation started to be visible seven days after flowering (DAF), increased rapidly from milky (11 DAF) to dough stage, then started decreasing after the peak was attained at 18 DAF. RNA-seq showed that 519 out of 14889, 477 out of 17914, and 1614 out of 18810 genes were uniquely expressed in the milky, doughy, and mature stages, respectively. We performed three pairwise comparisons: milky vs. dough, milky vs. mature, and dough vs. mature, and identified 6753, 9540, and 2531 DEGs, respectively. The DEGs' abundance was higher in milky vs. mature, with 5527 up-regulated genes and 4013 down-regulated genes, while it was smaller in dough vs. mature, with 1419 up-regulated genes and 1112 down-regulated DEGs. This result was consistent with the changes in anthocyanin profiling, and the expression of structural, regulatory, and transporter genes involved in anthocyanin biosynthesis showed their highest expression at the dough stage. Through the gene expression profile and protein interaction network, we deciphered six main contributors of the anthocyanin peak observed at dough stage, including Os*ANS, OsDFR, OsGSTU34, OsMYB3, OsbHLH015*, and Os*WD40-50*.

**Discussion:**

This study is the first to report the investigation of anthocyanin and gene expression at three developmental stages of black rice caryopsis. The findings of this study could aid in predicting the best harvesting time to achieve maximum anthocyanin content and the best time to collect samples for various gene expression analysis, laying the groundwork for future research into the molecular mechanisms underlying rice caryopsis coloration.

## 1 Introduction

Rice (*Oryza sativa* L.) is classified as the main staple food for more than half of the world’s population (Fukagawa and Ziska, 2019) and has become a political crop in the sense that its price and accessibility influence social stability (Arouna et al., 2021). Based on its color, rice can be classified as pigmented or non-pigmented. Natural pigmented rice is mainly black, purple, and red in color and contains a variety of phytochemicals (flavones, tannins, phenolics, sterols, and oryzanols) and essential oils (Pornngarm et al., 2019). Black rice is undoubtedly a special breed of rice that is cultivated on earth. In ancient China, it was reserved only for Chinese royalty and used as an ingredient in Chinese traditional medicines (Sati and Singh, 2019). At present, more than 200 types exist worldwide, distributed mainly in Southeast Asia and parts of Africa (Panda et al., 2022). China is the richest source of black rice, with more than 54 modern black rice varieties, holding the highest number of black rice accession numbers (359) in the germplasm collection, and producing about 62% of global black rice (Shinta et al., 2014; Kushwaha, 2016; Krishnan et al., 2020; Panda et al., 2022). Black rice emerges as a great source of antioxidants, ranking first among the four types of colored rice cultivars (black, purple, red, and brown) owing to its high amount of anthocyanin (Goufo and Trindade, 2014). These properties have raised research interest in agriculture, health science, and commerce significantly.

Anthocyanin is a plant secondary metabolite belonging to the flavonoid class with potential antioxidant properties beneficial to human health that sustains plant growth and development under different environmental stresses (Mackon et al., 2021a). Anthocyanins in rice caryopsis play different putative functions. Lately, with the rapid progress made in biotechnology, medicine, and molecular sciences, emerging scientific research, including animal models and human clinical trials, has highlighted diverse functions of anthocyanin in the human body, with the most prominent being antioxidant, anti-diabetic, anti-hyperlipidemic, anti-cancer, relieving inflammation, and increasing memory (Pornngarm et al., 2019; Shang et al., 2019; Krishnan et al., 2020). Therefore, endogenous knowledge advocates black rice for the treatment of chest pain, fever, gastrointestinal troubles, vomiting, diarrhea, wounds, hemorrhaging, and burns, as well as various liver and kidney disorders, rheumatism, paralysis, skin diseases, blood pressure, and leucorrhea (Hegde et al., 2013; Sathya, 2013; Mbanjo et al., 2020). Anthocyanins in black rice have economic value as pH indicators (Wahyuningsih et al., 2017), suitable candidates for dairy and ready-to-eat desserts, and food colorant substitutes (Turturica et al., 2015). The global anthocyanins market, valued at USD 291.7 million in 2014, was expected to reach USD 387.4 million by 2021 (Krishnan et al., 2020).

Anthocyanins in plants have been extensively studied in different model plant species like lisianthus (Zhang et al., 2006), grapevine (Gomez et al., 2011), arabidopsis (Poustka et al., 2007), rice (Mackon et al., 2021b), and other crops. Since black rice demand has increased in recent years, different studies have been conducted regarding anthocyanin in rice. In black rice, about 18 types of anthocyanins were identified among different cultivars (Goufo and Trindade, 2014; Mackon et al., 2021a). The types and amounts of anthocyanin vary between cultivars. The environmental growth and storage conditions may affect their concentration as well (Bakhshayeshi et al., 2006; Zhai et al., 2020). However, two main anthocyanins have been identified in black rice, namely cyanidin 3-glucoside and peonidin 3-glucoside, which account for 64–90% and 5–28% of total anthocyanins, respectively (Hou et al., 2013; Goufo and Trindade, 2014). Due to its methylated state, P3G is more stable than C3G (Hou et al., 2013). Anthocyanin can be accumulated in the stems, leaves, stigmas, apiculus, husk, and caryopsis with different intensities. Anthocyanin biosynthesis in rice is a common process that has been identified in other plants. Because anthocyanin can be synthesized differently in various parts of the plant, many studies have focused on the genetic basis of anthocyanin biosynthesis. However, limited studies have been carried out on the molecular mechanisms responsible for the caryopsis color in black rice and the expression of genes during grain maturation. Studies have highlighted that the synthesis of anthocyanin in rice is a branch of flavonoid pathways that start with phenylalanine as a substrate (Huang et al., 2019; Chen et al., 2021). Two groups of genes, structural and regulatory, contribute to these pathways. The first are structural genes, which encode the enzymes involved in the reaction. They are divided into two groups: early biosynthesis genes (EBGs) such as phenylalanine ammonia-lyase (PAL), chalcone synthase (CHS), chalcone isomerase (CHI), flavanone 3-hydroxylase (F3H), flavonoid 3’- or 3’-5’-hydroxylase (F3’H, F3’5’H), and late biosynthesis genes (LBGs) such as dihydroflavonol 4-reductase (DFR), anthocyanin synthase (ANS), leucoantho-cyanidin dioxygenase (LDOX), and uridine flavonoid-3-glucosyl transferase (UFGT). Although structural genes are required in the pathway to achieve anthocyanin synthesis, some studies found that some were difficult to correlate with anthocyanin because they are involved in other flavonoid biosynthesis, whereas others could be directly associated. For example, Lister and co-authors (1996) were not able to find a correlation between *PAL* and anthocyanin (Lister et al., 1996). Conversely, Reimold et al. (1983) pinpointed *CHS* as a key gene for anthocyanin synthesis (Reimold et al., 1983). Recently, Zhao et al. (2016) revealed that *CHS* expression was positively correlated with anthocyanin (Zhao et al., 2016). Other authors reported that the LBG are core genes for anthocyanin biosynthesis (Xia et al., 2021), and the mutation of *OsDFR, OsF3’H*, and *OsLDOX* induces non-pigmentation (Zhu et al., 2017; Jung et al., 2019). The second group are the regulatory genes, consisting of transcription factors (TFs), which regulate the activities of structural genes (Mackon et al., 2023). The major TF was identified as the complex R2R3MYB-bHLH-WD40 regulating anthocyanin in plants (Zheng et al., 2019; Xia et al., 2021). The *R/B* gene family coding for Myc bHLH protein and the *C1/Pl* gene family coding for Myb-type R2R3 were shown to control the deposition and accumulation of anthocyanin in black rice (Hu et al., 1996; Chin et al., 2016). Different plant species and cultivars have several homologs and paralogs of these TFs that regulate anthocyanin differently. MYB was described as the complex’s main component, directly targeting the gene promoter and controlling anthocyanin in specific parts (Mackon et al., 2023). Its expression pattern significantly affects the amount of anthocyanin in different parts of the plant (Mackon et al., 2021a; Xia et al., 2021). Although many MYB TFs were reported in rice, only three have been shown to regulate anthocyanin, namely *OsC1*, *Kala3*, and *OsPL* (Xia et al., 2021). Other studies reported coordinated sets of TFs that may differentially regulate anthocyanin biosynthesis in leaves, hulls, pericarps, and other tissues (Maeda et al., 2014; Oh et al., 2018; Sun et al., 2018; Mackon et al., 2021c). Once anthocyanins are synthesized in the cytoplasm, they are transported to the vacuole *via* several transporters. Three main transporter families have been found in different plants: glutathione-S-transferase (GST), multidrug and toxic compound extrusion (MATE), and the ATP binding cassette (ABC) family (Grotewold and Davies, 2008; Gomez et al., 2011; Mackon et al., 2021c).

With plant development, the pattern of anthocyanin accumulation varies both temporally and spatially within different species and cultivars. Anthocyanin synthesis and degradation occur concurrently, influencing coloration during plant development stages or environmental changes (Oren-Shamir, 2009). Sirijan et al. (2019) discovered that the concentration of anthocyanin in strawberries gradually increased from 15 to 30 days after anthesis (DAA) (Sirijan et al., 2019). Wu et al., 2019 found that the anthocyanin accumulation in red pears exhibited a rise-drop tendency from 15 to 115 days after full bloom, and the expression level of some genes such as *F3H, UFGT2, MYB10*, and *bHLH3* was correlated with the accumulation (Wu et al., 2019). Some studies revealed that planta degradation of anthocyanins may occur as a result of active enzyme-driven breakdown processes. For instance, a vacuolar class III peroxidase (*BcPrx01*) was suggested to be responsible for anthocyanin degradation in *Brunfelsia calycina* flowers, resulting in a rapid change from deep purple to completely white color after flowering (Vaknin et al., 2005; Zipor et al., 2015). In apples (*Malus domestica*), *MdCOP1* degrades the MdMYB1 protein, resulting in the inhibition of fruit coloration in a light-dependent manner (Li et al., 2012). Other studies focused on gene activity and discovered a link between key genes and the amount of anthocyanin at various stages of development. It was revealed that there is a correlation between anthocyanin accumulation and the gene network (Zou et al., 2019). Wang and co-authors showed that during the flower development stages of *Lycoris radiata*, the expression level of some genes (*LrDFR1, LsFLS*) was positively correlated with the anthocyanin content (Wang et al., 2021). Thill and his colleagues found that *F3’H* expression in *Fragaria ananassa* and *Fragaria vesca* plummeted as the fruit ripened. At all other stages, *F3’H* expression was high, which is consistent with anthocyanin (Thill et al., 2013).

Black rice caryopsis has gained increasing interest as a health-promoting food and is used in food and other industries. Because of its high protein and amylose content, indica rice is widely used to make rice noodles in southern China. However, research on the relationship between anthocyanin production and gene expression at different developmental stages in rice caryopsis is lacking. Such information is critical in assisting producers in determining the best harvesting time and maintaining high anthocyanin rice quality. In the present study, we used a new indica black rice variety with slender and long grains and studied the physiology and genetic characteristics of the caryopsis during grain development. We highlighted that anthocyanin accumulation in black rice is characterized by a gradual change in the color of the rice grain at different developmental stages. The pigment gradient increases as the rice grain develops and gradually fills. The color of the caryopsis starts to be visible 7-8 days after flowering (DAF), and the caryopsis becomes black rapidly, mostly at the milky stage. Further, the relationship between the amount of anthocyanin and gene expression was elucidated, and we deciphered the key genes during caryopsis development. The information obtained in this study could help predict the suitable harvesting time to achieve optimum health benefits. High-throughput sequencing has been recently revealed as a fast and reliable way to uncover differentially expressed genes (DEGs) in the biosynthesis pathway and different TFs related to specific traits in plants (Thill et al., 2013; Li et al., 2018; He et al., 2020; Li et al., 2020; Wang et al., 2021). We used both high-performance liquid chromatography (HPLC) and RNA-sequencing to study the anthocyanin profile and the dynamics of gene expression in rice caryopsis at different stages of development.

## 2 Material and methods

### 2.1 Plant material

**Figure 1:**
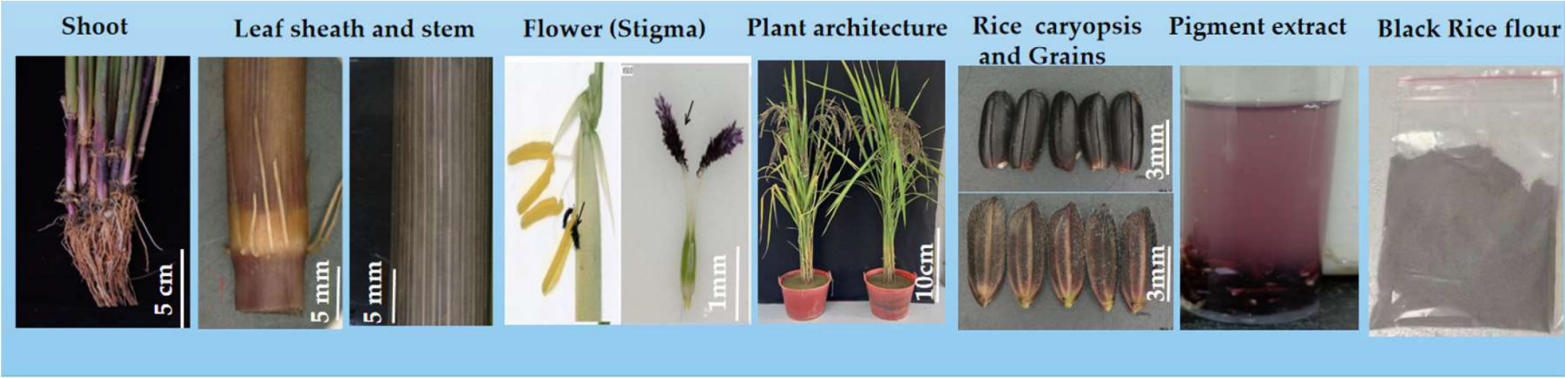
18BLN6321 black rice cultivar features; from left to right indicate the basal shoot, bar = 5 cm; stem with basal culm surrounded, leaf sheath at heading stage bar =5 mm; stigma at heading stage, the black arrows indicate inserted stigma, the scale bar = 1 mm; plant architecture, bar =10 cm at heading stage; black rice caryopsis, and endosperm at maturation stage, the scale bar = 3 mm; and black rice pigmentation extract.

An indica black rice cultivar, a true hybrid “18BLN6321” (Rong S/Y52052) with distinctive features, was utilized in this study. Considering that some black rice cultivars have only some tissues and organs that are pigmented, the cultivar used in this study is unique because anthocyanin was observed in almost all parts, including the caryopsis, leaf blade, leaf sheath, internodes, stem, ligules, apiculus, and stigmas (**Figure 1**). Our material was grown in the experimental field at Guangxi University during the autumn of 2021, which is the second rice-growing period of the year in Guangxi Province, Nanning, China (latitude: 22° 49’ 0.01” N, longitude: 108° 19’ 0.01” E). The yearly average temperature is 21.83°C (71.3°F), and there is 1190 mm of precipitation. The growing condition and all activities were carried out normally, as recommended in this area.

### 2.2 Methods

All data collection and observations were carried out from the blooming stage to the maturation stage. Before the flowering, we randomly selected and labeled about 100 plants with tags for the experiment. To avoid sample degradation, entire plants with root systems were collected and kept hydrated. Rice grains were collected from approximately five plants; the husk was removed, and the caryopsis were immediately frozen with liquid nitrogen and stored at -80°C.

#### 2.2.1 Grain investigation at developmental stages

Plants were collected from the field, and caryopsis were investigated at five developmental stages: 4 days after flowering (DAF), 11 DAF, 18 DAF, 25 DAF, and 35 DAF, representing flowering, milk grain, dough grain, mature grain, and ripening stages, respectively. A super-depth three-dimensional (3D) microscopic imaging system was used for photography and processed further using Adobe Photoshop 2022 (Adobe, http://www.adobe.com). Other parts of the plant were investigated as well.

#### 2.2.2 Anthocyanin quantification through HPLC

We identified and quantified anthocyanin in rice samples using HPLC (Palapol et al., 2009; Sirijan et al., 2020). To precisely measure the anthocyanins at four developmental stages, three anthocyanin standards were used, including cyanidin-3-O-glucoside chloride, peonidin-3-O-glucoside chloride, and petunidin-3-O-glucoside chloride. The procedure was as follows: Briefly, 1 g of freeze-dried rice caryopsis was pre-treated with 50% alcohol containing 1% formic acid. Following pre-treatment, samples were vortex-mixed for 30 seconds and sonicated for 30 minutes. After centrifugation for 30 minutes at 13000 rpm, 0.45-mm nylon mesh was used to filter the supernatant. The analysis was performed by injecting a 30 μL aliquot of samples through the membrane of an HPLC column. Solutions of (A): acetonitrile + 0.1% formic acid and (B): acetonitrile/water/formic acid (5:94.9:0.1) were used as the mobile phases. The compounds were separated on reversed-phase, 250 mm × 4.6 mm i.d., 5 m columns. Waters XBridge C18 with 1% formic acid and acetonitrile was used as the mobile phase. Elution was processed with two solvents. Solvent A contains 1% formic acid in water, whereas solvent B contains 1% formic acid in acetonitrile. The gradient program of solvent A was 0% at zero time and ramped linearly to 20% at 20 min, 30% at 26 min, 50% at 28.5 min, 50% at 28.5 min, 95% at 32 min, and back to 0% at 35 min. The gradient profile for the separation of anthocyanin was 92% A-8% B (0 min). This profile gradually changed linearly to 20% A-80% B for 20 minutes, was maintained for 2 minutes, and then returned to 92% A–8% B, which was maintained for 8 minutes. The monitoring system was performed using a 520-photodiode array detector (PDA); the temperature of the column and flow rate were 35°C and 0.8 mL/min, respectively. All chromatograms were recorded at 530 nm. By comparing the chromatograms to external standard calibration curves, anthocyanins were quantified as mg/kg fresh weight (FW), and the experiment was repeated three times.

#### 2.2.3 Transcriptome analysis

##### 2.2.3.1 cDNA library construction, sequencing, and transcriptome assembly

To perform RNA-sequencing, 0.5 g of rice samples at different developmental stages were ground in the mortar under liquid nitrogen freezing conditions and added to the eppendorf tube. For total RNA isolation, we used the TRIzol^®^ reagent kit (Invitrogen, Carlsbad, CA, USA) according to the manufacturer’s instructions. RNA quality and quantity were double-checked. A Nanodrop 2000 spectrophotometer was used to measure the concentration, and an Agilent 2100 was used to measure the RNA integrity number (RIN) and 1% agarose gel electrophoresis was used to measure the integrity. Samples having RIN ≥ 7 were used for cDNA library synthesis using a TruSeqTM RNA sample preparation kit (San Diego, IL, CA, USA). Briefly, mRNA was isolated and enriched from the total RNA using oligo (dT)-attached magnetic beads. Then, mRNA was fragmented into a small sequence of 300 bp and transcribed into first-strand cDNA, followed by second-strand synthesis using reverse transcriptase (Invitrogen, CA, Carlsbad, USA) and stabilized with the random hexamer primer (Illumina). The purified double-strand cDNA was subjected to 3’ sticky end A base terminal repair and prepared for hybridization. Repaired cDNA was amplified by PCR for 15 cycles, separated on 2% certified low-range ultra-agarose (Bio-Rad, Hercules, CA, USA), and quantified by TBS-380 Picogreen (Invitrogen, Carlsbad, CA, USA). Amplified fragments were subjected to deep sequencing by the Illumina Hiseq Xten/NovaSeq 6000 sequencer platform, performed by Shanghai Majorbio Bio-pharm Technology Corporation (Majorbio Bio-pharm, Shanghai, China).

##### 2.2.3.2 Data filtering and mapping of reads

The quality of the original sequencing data was pre-processed by removing adapters, low-quality reads, and low-quality bases using the trimmatic software SeqPrep (http://github.com/jstjohn/SeqPrep; accessed on December 7, 2021) and Sickle (http://github.com/najoshi/sickle; accessed on December 7, 2021) with default parameters and assembling the high-quality reads. Then, clean reads were aligned to the O. sativa japonica reference genome IRGSP-1.0 (http://plants.ensembl.org/Oryza_sativa/Info/Index; accessed on December 5, 2021). We used Hisat2 (http://ccb.jhu.edu/software/hisat2/index.shtml; accessed on December 10, 2021) or TopHat2 (http://tophat.cbcb.umd.edu/; accessed on December 10, 2021) (Kim et al., 2015) to compare clean reads with the reference genome and obtain position information (map data) on the reference genome, as well as the characteristics of the specific sequence of the sequencing samples. StringTie used a reference-based approach to assemble clean reads (https://ccb.jhu.edu/software/stringtie/index.shtml?t=example; accessed on December 10, 2021) in a reference-based approach (Pertea et al., 2015).

##### 2.2.3.3 Functional annotation and enrichment analysis

Alignment of the assembled unigenes was performed and annotated against the public databases, including Gene Ontology (GO) databases, Swiss-Prot protein databases, non-redundant (Nr) protein and nucleotide (Nt) databases, Protein Family (Pfam) databases, Cluster of Orthologous Groups (COG) databases, and the Kyoto Encyclopedia of Genes and Genomes (KEGG) database, to get the functional annotation of DEGs’ E-value cut-off (E < 1 × 10^−5^). A GO enrichment analysis of transcripts in the gene set was performed using the software Goatools 0.6.5 (https://github.com/tanghaibao/GOatools; accessed on December 5, 2022) (Tang et al., 2015), and transcripts were considered significantly enriched when the p-value (FDR) ≤ 0.05.

##### 2.2.3.4 Differential expression functional enrichment analysis

The expression level of unigenes was analyzed in different samples at different developmental stages. Gene abundances were quantified through RNA sequence by expectation maximization (RSEM) (http://deweylab.biostat.wisc.edu/rsem/; accessed on December 20, 2022) (Li and Dewey, 2011). We used transcripts per million reads to normalize the expression value of reads. The differential expression between samples was performed using DESeq2 with pairwise comparison (Love et al., 2014). The testing criterion was set based on multiple hypotheses, with the *p*-value threshold, false discovery rate (FDR ≤ 0.05), and (log2FoldChange ≥ 1) considered significantly different.

##### 2.2.3.5 Transcription factor analysis and protein interaction prediction

The advanced analytics tools in the transcription factor PlantTF database version 3.0 were used to predict which transcription factors are involved in the formation of color in the caryopsis. We also looked into the expression of TFs involved in the flavonoid biosynthesis pathway, such as MYB, bHLH, and WD40, using the blastx software with an e-value of 1e-10. A Hmmscan e-value of 1e-10 was used to analyze the transcription factor family to which the gene belongs and construct the heatmap. Using the network modeling method, a set of genes related to the biosynthesis of anthocyanin, including structural, regulatory, and transporter DEGs, was used to build a network of how proteins interact with each other. From the level of the network in the string database, the topological attribute analysis of the network was done, and the complex biological data could be used to find out how important proteins interact with each other. Transitivity was used to measure the network correlation. Cytoscape version 3.6.1 was used to make a picture of the interaction network between structural, regulatory, and transporter DEGs.

#### 2.2.4 Expression analysis by quantitative reverse transcription PCR

We used qRT-PCR to assess gene expression and confirmed the RNA-Seq results. Following the manufacturer’s instructions, the total RNA was extracted from rice caryopsis using the TRIzol^®^ Reagent Kit (Invitrogen, Carlsbad, CA, USA). We selected eight important genes, including one EBG (*OsF3’H*), three LBGs (*OsANS, OsDFR*, and *OsGT*), three transcription factors (*OsMYB3, OsKala4*, and *OsWD40-50*), and one transporter (OsGSTU34), that were differentially expressed at developmental stages in the caryopsis. In order to standardize the amount of gene expression, rice *OsActin1* was employed as an internal reference gene. The primer sequences obtained from the database https://biodb.swu.edu.cn/qprimerdb/ (accessed February 2, 2023) are enlisted in the **Supplemental Table 1**. Following the manufacturer’s instructions, the primeScriptTM RT reagent kit with gDNA eraser (RR047A, Takara, Japan) was used to create first-strand full-length cDNAs from 2 g of total RNA. According to the manufacturer’s instructions, qRT-PCR was performed on the LightCycler 480II (Roche) using the ChamQTM SYBR qPCR Master Mix (Q311-01, Vazyme, China). In order to create gene expression profiles, three biological repetitions were employed. The reaction was modified to match the thermal cycling conditions, which were 95°C for 30 seconds during the first denaturing phase, followed by 40 cycles with 95°C for 5 seconds and 60°C for 20 seconds in each cycle. The 2^−ΔΔCt^ calculation technique was used to determine the gene expression level.

## 3 Results

### 3.1 Color changes during caryopsis development as a visual sign of anthocyanin accumulation

**Figure 2:**
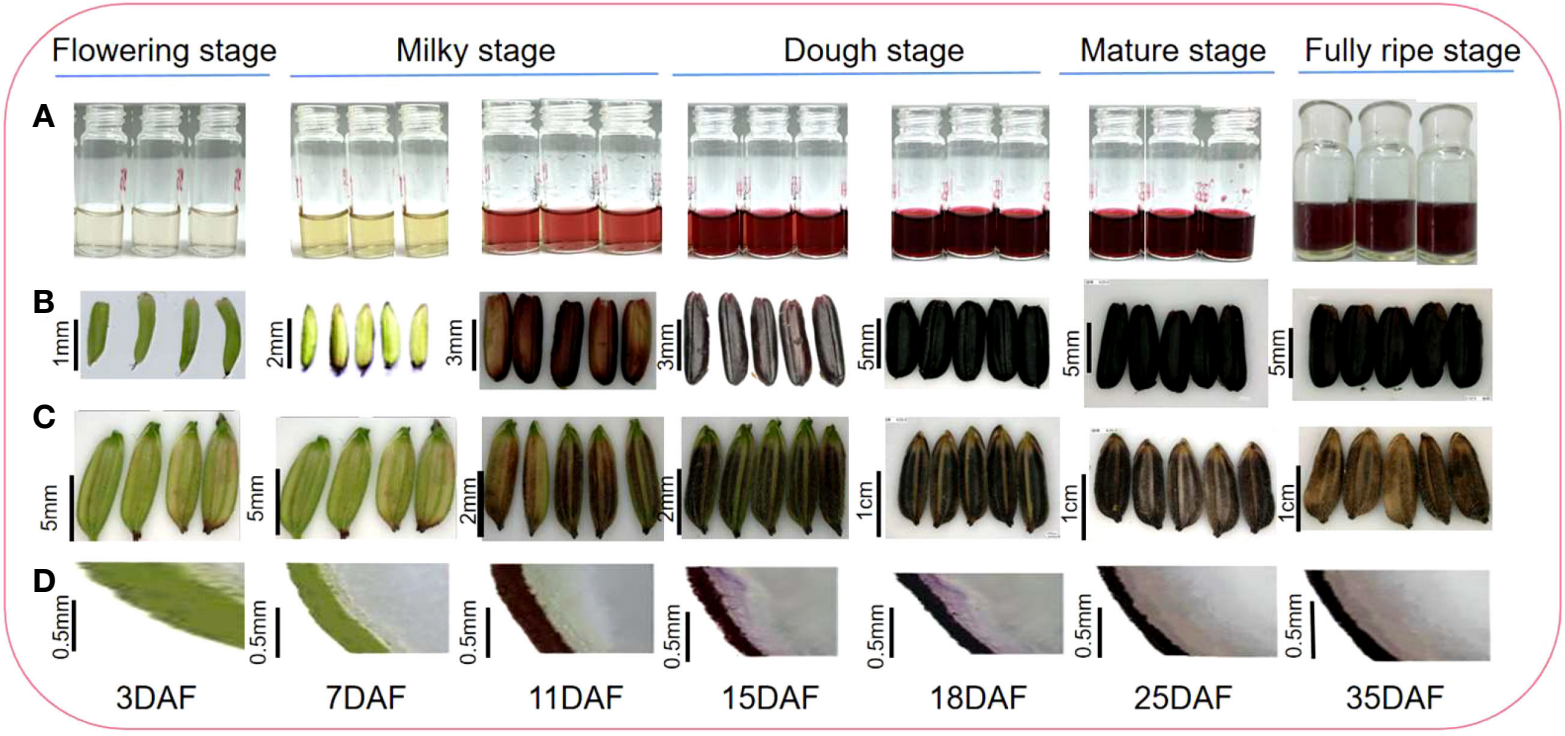
Black rice grain developmental stage and anthocyanin accumulation; **(A)** anthocyanin extract; **(B)** rice caryopsis; **(C)** rice grain; **(D)** rice section.

The rice caryopsis underwent rapid and gradual changes at different development stages, from the flowering stage to full maturation (**Table 1**). During the first five days after the anthesis, called the flowering stage, the caryopsis was completely green. This color began to change at the milky stage (7 DAF), and by 11 DAF, nearly half of a caryopsis part was colored purple, but the intensity of the color was not strong. At the dough stage (15–20 DAF), the caryopsis is fully colored and looks dark purple. As the caryopsis matures to the fully ripe stage, the intensity increases and it appears darker (**Figure 2**). Indeed, the coloration was not uniform for all grain, but for about 80% of grain, the color was similar. The gradual change may be the result of anthocyanin accumulation in the caryopsis.

**Table 1:** Description of grain development stage of black rice.

Development stage	Day after flowering	Hull color	Caryopsis color and form	Endosperm texture
Flowering	0-5	Green with purple apiculus	Green, curvy and filiform	Empty without endosperm.
Milky	6-14	The hull is turning purple	Soft, uniform, and light-dark	Milky, white endosperm.
Dough	15-21	The hull is light-dark	Soft, elongated, and darker	Dough, thickening, gray endosperm
Mature	22-30	The grain is mature and hard, and the hull is dark in color	The caryopsis is tender and dark in color.	Crystallized and darker.
Fully ripe	31-40	Fully mature, hard and brownish	Hard, large, uniform and black dark	Fully crystallized, with uniform dark color

### 3.2 Anthocyanin quantification at different developmental stages

**Figure 3:**
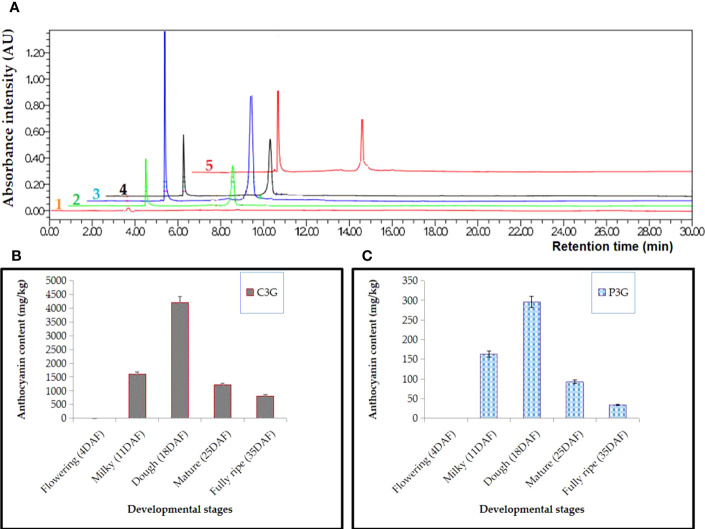
HPLC analysis in black rice caryopsis; **(A)** Anthocyanin absorbance for different samples, 1 refers to flowering stage, 2, milky stage, 3, dough stage, 4, mature stage, 5, fully mature; **(B)** anthocyanin content cyanidin-3-O-glucoside (C3G); **(C)** anthocyanin content peonidin-3-O-glucoside (P3G) at different stage; Anthocyanin content is represented as value ± SD of three biological replicates; DAF, days after flowering.

High-performance liquid chromatography (HPLC) was used to measure the amount of C3G and P3G, the two main anthocyanins in black rice, in triplicates at five stages of growth: flowering, milky, dough, mature, and fully ripe. Anthocyanin could not be detected at the early stage (3-5 DAF), according to the results. The anthocyanin started to be detected at about 8 DAF and increased from 10 DAF to 20 DAF, during which the peak was obtained mainly at the dough stage. At peak, the concentration of anthocyanin was 4206.91 ± 18 mg/kg of fresh weight (FW) and 295.33 ± 4 mg/kg FW for C3G and P3G, respectively. The amount of anthocyanin decreased by about 5 times between 20 and 35 DAF. The concentration of P3G was 295.33 ± 13 mg/kg FW, 97.52 ± 10 mg/kg FW, and 35.98 ± 7 mg/kg FW, while the C3G concentration was 4206.91 ± 118 mg/kg FW, 1212.87 ± 60 mg/kg FW, and 814.59 ± 35 mg/kg FW at the dough, mature, and fully ripe stages, respectively. Remarkably, the amount of C3G was 10 times higher than that of P3G. In C3G, it varies between 91 and 93% of TAC, whereas in P3G, it was approximately 7–9% of TAC. For all samples, the peak was obtained at the dough stage, while the lowest concentration was obtained at the fully ripe stage just before the rice was harvested (**Figure 3**).

### 3.3 Overview of sequencing and transcriptome assembly

**Figure 4:**
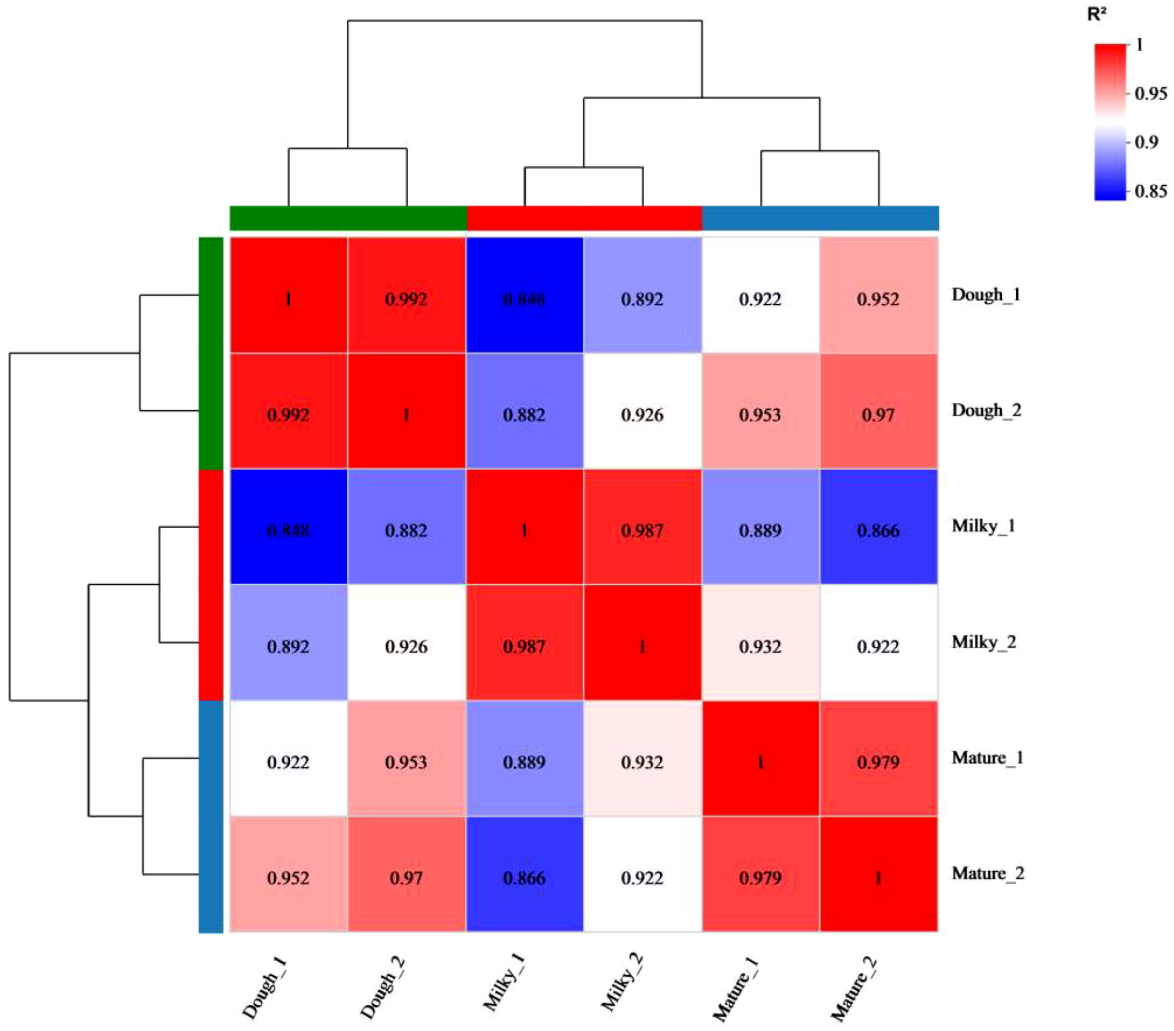
Correlation analysis between different samples.

To get a global view of the black rice transcriptome at three different stages of development—milky, doughy, and mature—a sample was taken and used to make six DNA libraries, which were then sequenced with high-throughput Illumina Hiseq Xten and Nova. The RNA sequencing yielded 44.23–57.72 million (M) of raw data in total, with 150 bp for both paired ends. After adaptor removal and data filtration, 6.4-8 Gbp of clean base and 43.81–57.13 million (M) paired-end clean reads from each sample with Q30 percentages > 95.8% and GC percentages > 51% were obtained, respectively. The Pearson correlation analysis and hierarchical cluster revealed that the correlation coefficient between samples was different and greater than 0.84 (**Figure 4**). This finding supported the sequencing correlation and suggested that developmental stage has a significant impact on transcriptome profiles. Subsequently, the principal component analysis showed that replicated samples were relatively close, while the distance between samples of different stages was high (**Supplemental Figure 1**). This result highlighted the genetic similarity and difference between samples and cultivars, respectively, at different stages.

The mapping ratio of mapped reads to the Japonica rice genome fell into a range of 94.9 and 95.2% (**Table 2**). More than 79% of the aligned reads from each sample mapped to exonic regions of the reference genome, 0.8-1.2% mapped to intergenic regions, and 0.8-1.8% mapped to intronic regions (**Supplemental Table 2**). The majority of the reads were distributed on chromosome 2, with a lower read distribution on chromosome 11.

**Table 2:** Summary of RNA seq data.

Sample ID	Clean base	Raw Reads	Clean Reads	Total Mapped Reads	Q20(%)	Q30(%)	GC (%)
Milky-rep1	6391590189	44487444	43814814	41492963(94.7%)	98.79	96.27	51.4
Milky-rep2	6921123625	47527712	46925570	44457493(94.74%)	98.78	96.17	51.67
Dough-rep1	8018170634	54482624	54089936	51460136(95.14%)	98.8	96.21	52.01
Dough-rep2	7935042742	54430818	53880872	51159662(94.95%)	98.71	95.99	52.25
Mature-rep1	8413306716	57726156	57135956	54078567(94.65%)	98.78	96.23	54.57
Mature-rep2	7312460913	49975758	49497564	46688293(94.32%)	98.78	96.21	54.56

Rep1 and Rep2 indicate respectively replication 1 and replication 2 at different development stage; milky = sample collected at 11 days after flowering; dough = samples 18 days after flowering; Maturity = sample at 25 days after flowering; Clean reads: total number of entries of sequencing data after quality control; clean bases: total number of sequencing data after quality control; GC%, represents Guanine-Cytosine content which is often rich in the coding region and calculated as count [(G + C)/count(A + T + G + C)] × 100; Q20 indicates the probability of an incorrect base call is 1 in 100; Q30, 1 in 1000 and their value refer respectively to the percentage of bases with sequencing quality above 99% and 99.9%.

### 3.4 Identification of differential expressed genes (DEGs)

**Figure 5:**
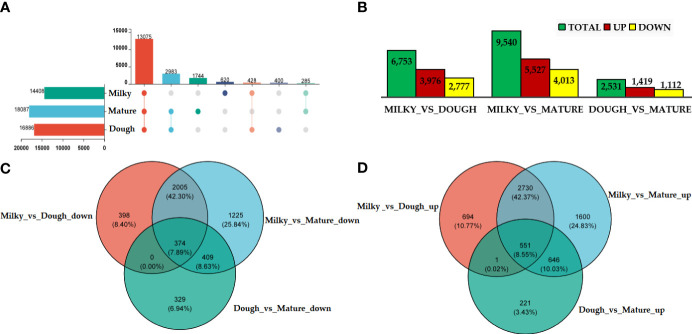
DEGs analysis at different stage; **(A)** upset of DEG; The left horizontal column chart represents the element statistics of each set, in the middle matrix, a single point represents an element unique to a set, the connection between points and points represents the intersection unique to different sets, and the vertical column chart represents the corresponding intersection element values; **(B)** Statistical table of the number of differential genes; the abscissa represents the different comparison groups, and the ordinate represents the corresponding number of up-and down-regulated genes; **(C)** Venn diagram of down-regulated DEGs; **(D)** Venn diagram of up-regulated DEGs.

Gene expression was analyzed in each group. The results revealed that 13711 were commonly expressed in different groups. We identified 18087, 16886, and 14408 DEGs in the mature, doughy, and milky stages, respectively (**Figure 5A**). Further, uniquely expressed gene analysis showed 519 out of 14889, 477 out of 17914, and 1614 out of 18810 genes, respectively, in the milky, doughy, and mature stages (**Supplemental Figure 2**). Based on the quantitative results of expression, differential expression analysis of genes between groups was applied using Deseq2 log2FoldChange ≥ 1 and the p-value cut-off (FDR ≤ 0.05). At different development stages, thousands of DEGs were found. We performed three pairwise comparisons: milky vs. dough, milky vs. mature, and dough vs. mature, and identified 6753, 9540, and 2531 DEGs, respectively. In all comparisons, the number of up-regulated genes was higher than the number of down-regulated genes. The DEGs' abundance was higher in milky vs. mature, with 5527 up-regulated genes and 4013 down-regulated genes, while it was smaller in dough vs. mature, with 1419 up-regulated genes and 1112 down-regulated DEGs (**Figure 5B**). We further screened the overlap of DEGs among all comparison groups. A total of 374 significantly down-regulated and 551 significantly up-regulated genes were commonly expressed in all comparisons at different developmental stages (**Figures 5C, D**). Some of these genes may be associated with the regulation of caryopsis color.

### 3.5 Functional annotation and enrichment analysis

**Figure 6:**
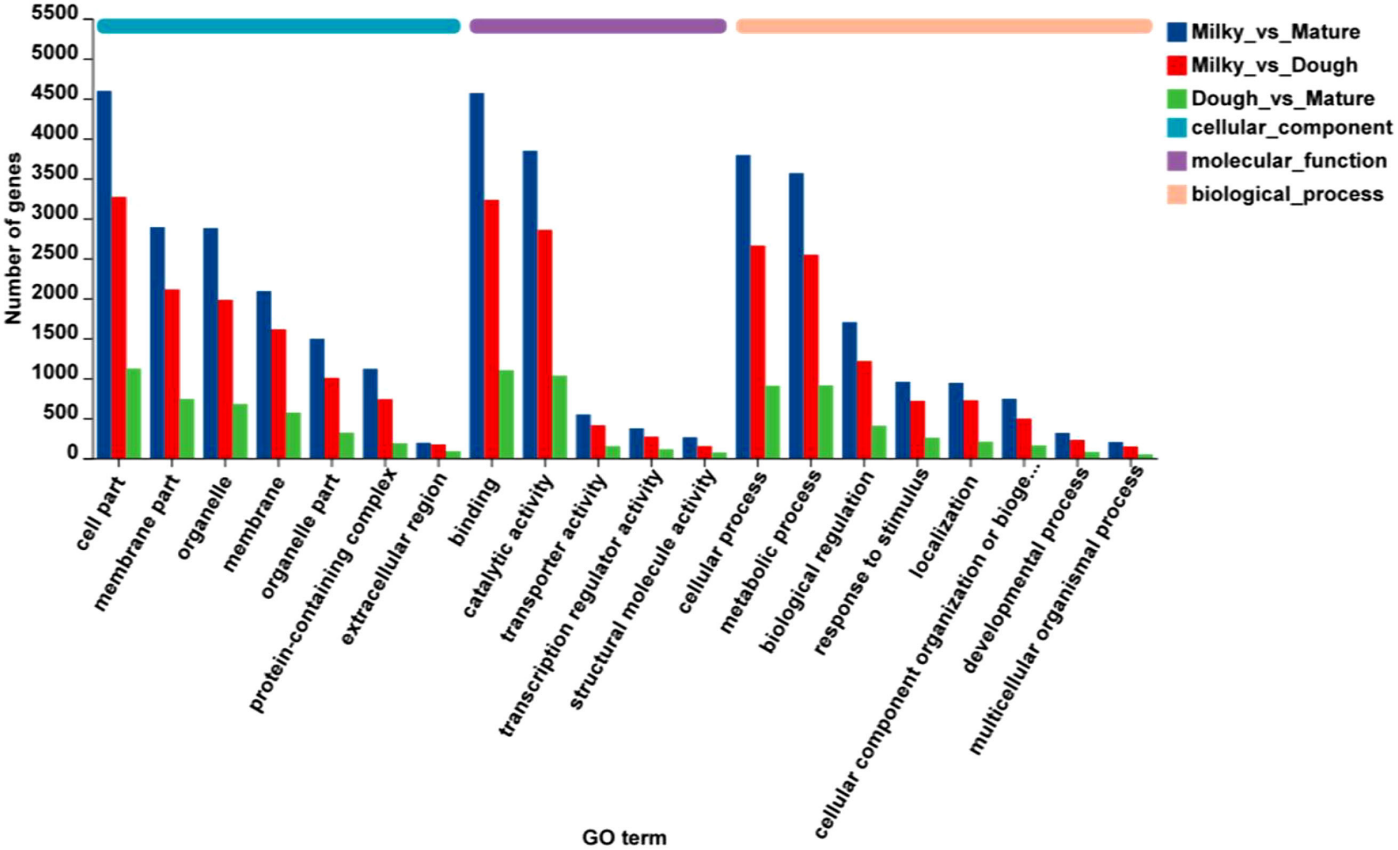
Gene ontology classification; The abscissa represents in the figure represents the secondary classification term of GO, the ordinate the number of genes/transcripts in the secondary classification on the comparison, and the color indicates different gene sets.

Functional annotation was performed through a BLAST search of all unigenes against the public databases. 27523, 12099, 29107, 32580, 23472, and 21009 unigenes were found to be annotated to the GO, KEGG, COG, NR, Swiss-Prot, and Pfam databases, respectively. To characterize the DEGs, GO and KEGG were carried out in different comparisons, including milky vs. dough, milky vs. mature, and dough vs. mature. The result showed for all comparisons that two cellular components, including cell part, membrane part, and organelle, were higher; in molecular function, binding and catalytic activity were high; and in biological process, cellular process, metabolic process, and biological regulation were high (**Figure 6**). GO enrichment analysis was used in three main categories: biological processes, molecular function, and cellular component, to try to figure out what the biological function of DEGs is.

**Figure 7:**
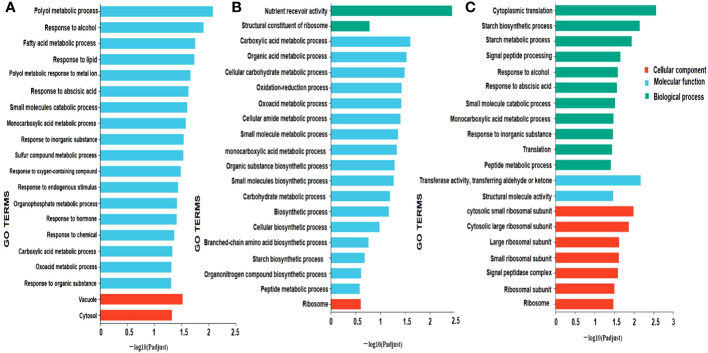
GO enrichment classification. **(A)** milky vs. Dough; **(B)** Dough vs. Mature; **(C)** milky vs. Mature. The ordinate represents the GO term, and the abscissa represents the significance level of enrichment, corresponding to the height of the column, where the smaller the FDR, the larger the -log10 (P-adjust) value, the more significant the GO term. The three colors represent three major classifications, namely biological processes (BP), cellular components (CC), and molecular functions (MF). By default, the enrichment results of top20 are displayed under the premise of P-adjust <0.5.

The biological function of the DEGs was predicted using GO enrichment analysis. This was done in three main areas: biological process, molecular function, and cellular component. The results showed that GO enrichment was different for each comparison (**Figure 7**). In milky vs. dough, cellular components (vacuole, cytosol) and molecular function were significantly enriched. In Dough vs. Mature, the biggest DEGs were enriched in molecular function, especially metabolic and biosynthesis processes, and biological processes (the activity of the nutrient reservoir). No GO was enriched in biological processes. In milky vs. mature, DEGs were enriched in biological processes (cytoplasmic translation, starch biosynthesis, and metabolic processes), cellular components (ribosomal subunits), and molecular functions (transferase and structural molecule activity).

**Figure 8:**
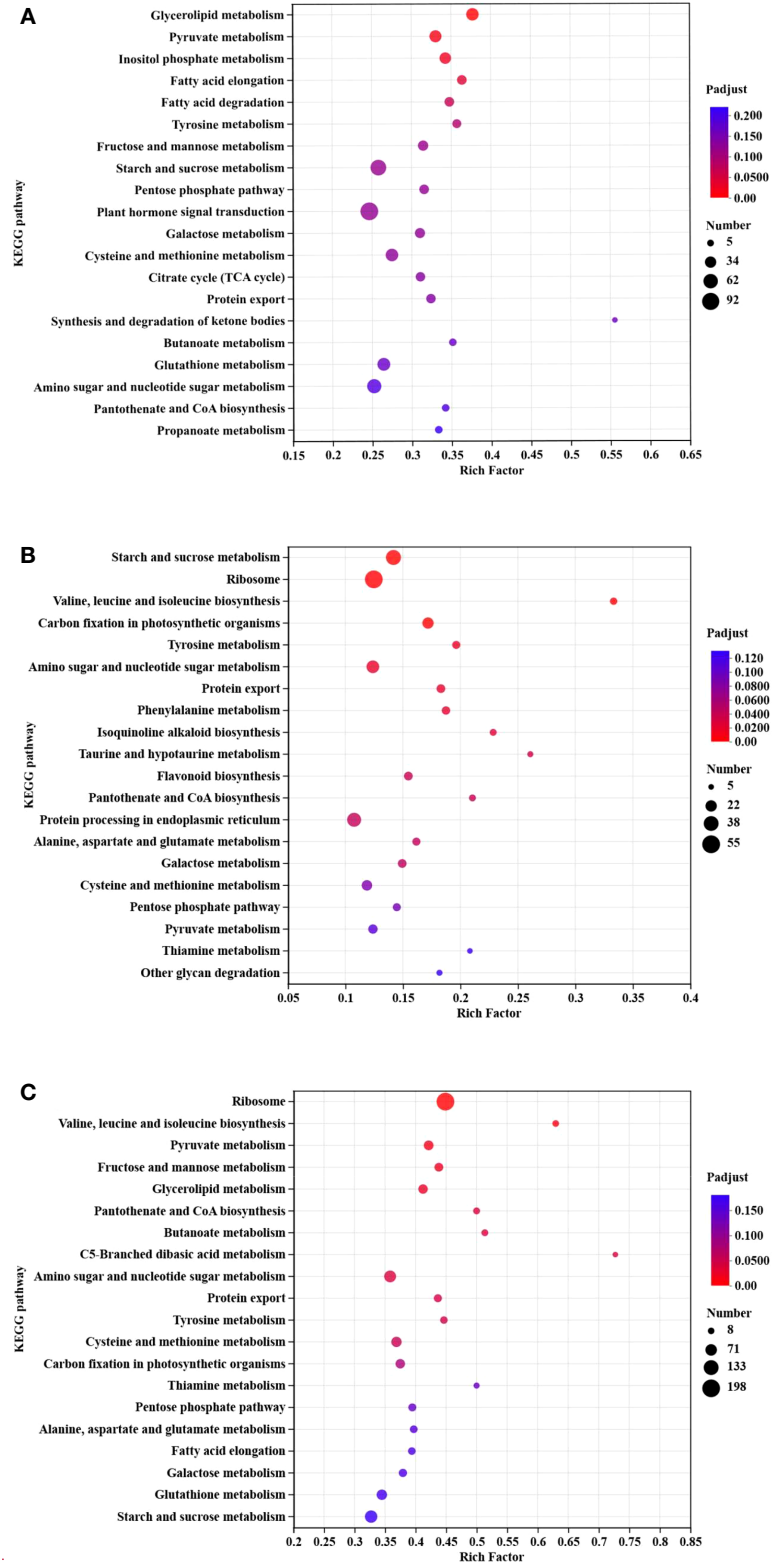
KEGG enrichment classification. **(A)** milky vs. dough; **(B)** dough vs. mature; **(C)** milky vs. mature. The enrichment results of the TOP20 are displayed under the premise of *p*-adjust<0.5. The vertical axis represents the pathway name, and the horizontal axis represents the ratio of the number of genes enriched in the pathway to the number of annotated genes. The larger the rich factor, the greater the degree of enrichment, the size of the dot indicates the number of genes in this pathway, and the color of the dot corresponds to different padjust ranges.

KEGG pathway enrichment was carried out for different comparisons: milky vs. dough, dough vs. mature, and milky vs. mature (**Figure 8**). Based on our results, the pathways with the most genes were glycerolipid metabolism (Map00561) and pyruvate metabolism (Map00620), which had 40 and 43 genes, respectively. Most DEGs were enriched in lipid and carbohydrate metabolism. In Dough vs. Mature, starch and sucrose metabolism (Map00500) were the most enriched pathways, followed by ribosomes (Map03010), which contain 39 and 55 genes, respectively. Besides, we found that among the top 20 pathways, phenylalanine metabolism (Map00360) and flavonoid biosynthesis pathways (Map00941) were highly enriched. Moreover, flavonoid biosynthesis pathways contained 13 genes. The DEGs in these enriched pathways appeared to be associated with the upstream regulation of anthocyanin biosynthesis. In the milky vs. mature comparison, the ribosome pathway (Map03010) was remarkably abundant, containing 198 genes, followed by valine, leucine, and isoleucine biosynthesis with only 17 genes. Overall, most DEGs were assigned to sugar, lipid, amino acid, and transport activities. In addition, all comparisons included genes related to flavonoid biosynthesis, although these were less enriched in milky vs. dough and dough vs. mature. Likewise, other genes related to transport activity and transcription factors were identified.

### 3.6 Characterization of functional genes responsible for the anthocyanin biosynthesis pathways

Anthocyanin biosynthesis is a part of the flavonoid biosynthesis process. It is made up of structural genes that code for enzymes, regulatory genes that control the expression of structural genes, and transporter genes that store the anthocyanin

#### 3.6.1 Identification of key structural genes differentially expressed during caryopsis development

**Figure 9:**
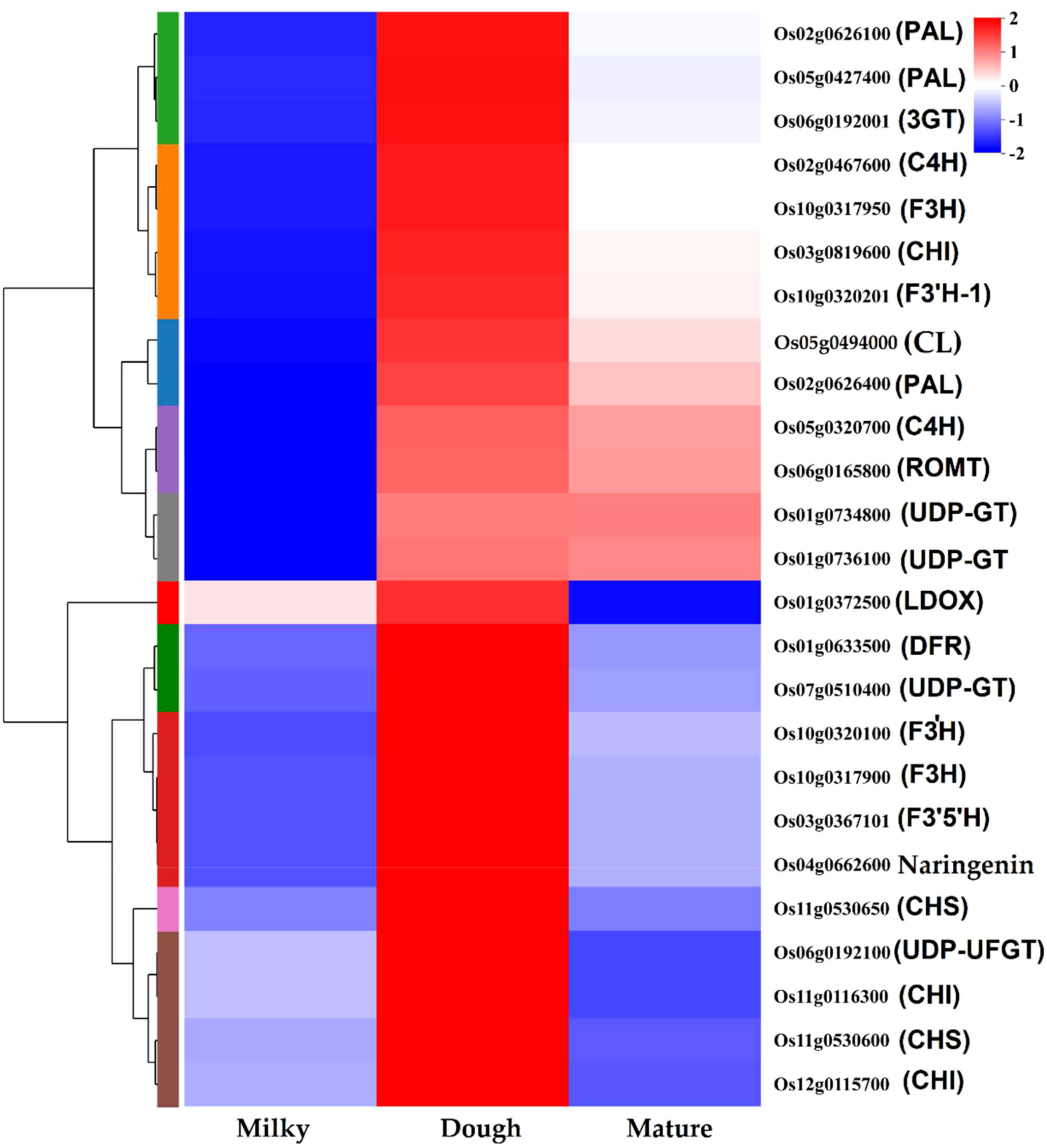
Heatmap of anthocyanin structural genes.

We investigated DEGs at different developmental stages of black rice caryopsis to identify the molecular basis underlying the difference in anthocyanin content. The anthocyanin biosynthesis could be divided into three phases: the phenylalanine biosynthesis genes in which (*PAL)*, the flavonoid biosynthesis genes (*C4H, 4CL, CHS, CHI, F3H, F3′H, DFR, ANS, FLS, ANR*, and *LAR)*, and the decorative genes involved in the conversion of anthocyanidin to anthocyanin, mainly *UFGT*. Our findings revealed a total of 83 DEGs that are potentially involved in anthocyanin. Among these genes, nine belonged to phenylalanine biosynthesis, 71 were found in the flavonoid pathway, and three were involved in the conversion of anthocyanidin to anthocyanin (**Supplemental Table 3**). Furthermore, a Pearson’s correlation coefficient was calculated between the expression levels of these DEGs in different samples. The results revealed that 39 genes among the 83 were significantly regulated in all comparisons (**Supplemental Table 4**). The number of significant DEGs was abundant in dough vs. mature, with the former containing 26 DEGs, with four up-regulated and 22 down-regulated. In milk vs. dough, 18 DEGs were identified, and all were upregulated. In milky vs. mature, 16 genes were found, containing 15 up-regulated genes and 1 down-regulated gene. The heatmap showed that these genes could be divided into two main clusters (**Supplemental Figure 3**). In cluster I, gene expression increased with the developmental stages, from milky to dough to mature. In the dough stage, no genes were significantly expressed, but they were highly expressed in the mature stage. We speculated that these genes may not be directly related to anthocyanin synthesis but may be involved in seed maturation. In cluster II, the expression trends were similar to the anthocyanin content. We obtained 25 structural genes that may contribute to anthocyanin biosynthesis after filtering those genes in Cluster I (**Figure 9**).

#### 3.6.2 Identification of regulatory genes involved in anthocyanin biosynthesis

**Figure 10:**
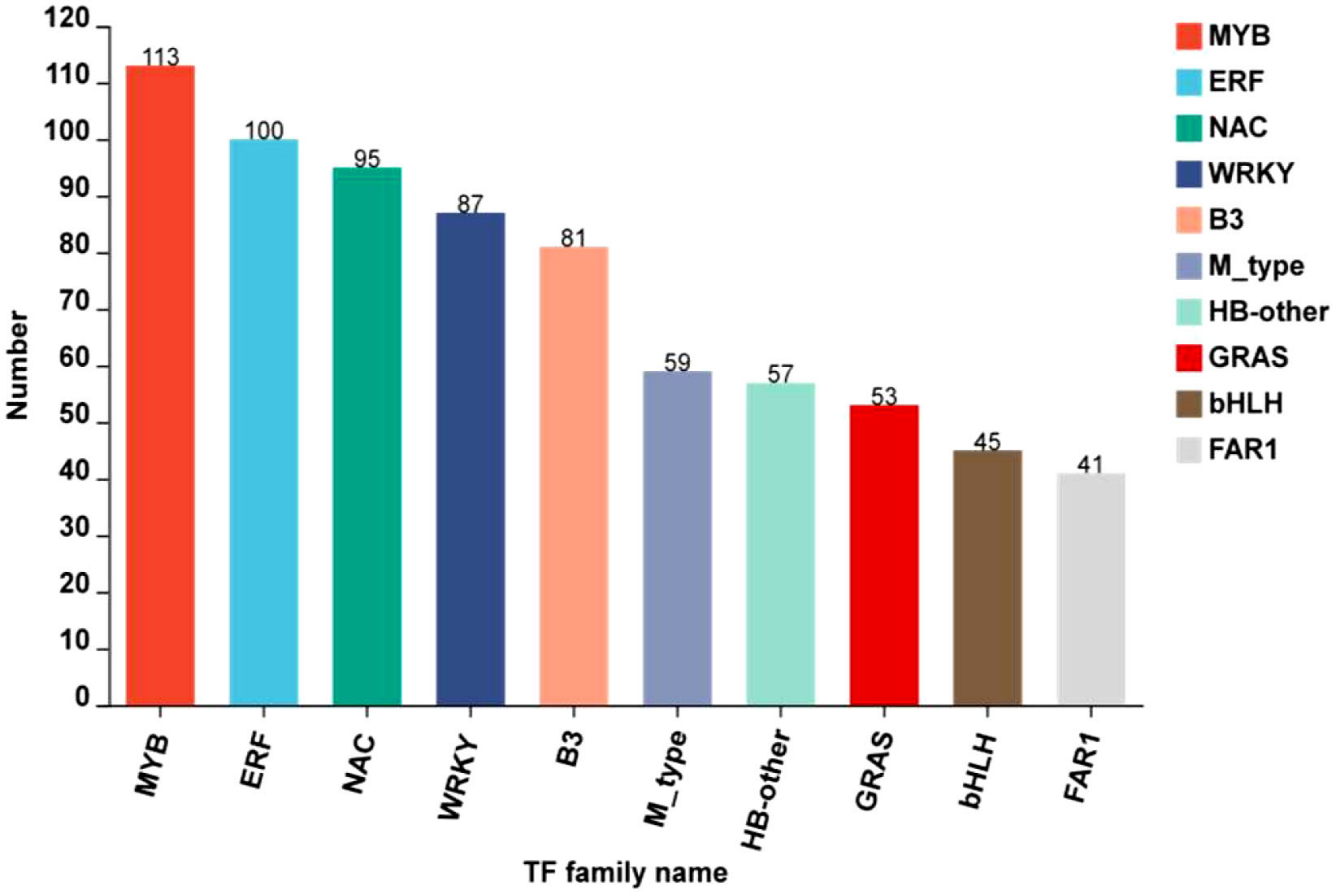
TOP10 of transcription factors families identified in black rice caryopsis.

At developmental stages, transcription factors modulating anthocyanin accumulation and biosynthesis in rice caryopsis were predicted. The results showed a total of 1236 TFs by searching through plantTFDB (e-value < 1.0E-10). The classification revealed that most TF belonged to 10 families, including MYB, ERF, NAC, WRKY, B3, M-type, HB-other, GRAS, bHLH, and FAR1, with MYB as the most abundant (**Figure 10**).

Most of the significantly regulating TFs were expressed in milky vs. mature (422), milky vs. dough (328), and less in dough vs. mature (168). Previous studies reported that anthocyanin is regulated by the complex R2R3MYB-bHLH-WD40 (Zheng et al., 2019; Mackon et al., 2021a; Xia et al., 2021; Mackon et al., 2023). We further screened MYB, bHLH TFs, and the WD40 gene family and found in the MYB TF family 47, 45, and 19 DEGs; and in the bHLH TF 17, 9, and 7 DEGs, respectively, in milky vs. mature, milky vs. dough, and dough vs. mature. Some TFs were shared among those comparisons, and after filtration, we found a total of 62 MYBs (**Supplemental Table 5**) and 19 bHLH (**Supplemental Table 6**). The same analysis was performed for WD-40, and the result revealed 51 DEGs significantly expressed in different comparisons, and 37 DEGs after filtration were identified (**Supplemental Table 7**). To identify putatively expressed DEGs of anthocyanin, we carried out heatmaps at different developmental stages (**Supplemental Figures 4A-C**). The result showed that 11MYB TFs, 3 bHLH TFs, and 7WD40 expression were consistent with anthocyanin accumulation, suggesting that these genes may be associated with high anthocyanin biosynthesis (**Table 3**).

**Table 3:** Differential regulatory genes (MYB-bHLH-WD40) based on fold-change levels for the three developmental stages.

Gene_id	Gene name	Log2FC Dough/Milky	Log2FC Mature/Dough	Log2FC Mature/Milky	Gene description
MSTRG.13983	–	3.45	-1.92	–	–
MSTRG.19976	–	2.34	-2.53	–	–
*Os01g0685400*	*-*	2.02	-1.52	–	Homeodomain-like domain containing protein
*Os05g0553400*	*OsMYB55*	5.16	–	4.78	Similar to Myb-related transcription factor-like protein (MYB transcription factor)
*Os08g0549000*	*OsGL1A*	3.26	–	2.42	R2R3 MYB transcription factor, Homologue of Arabidopsis transcription factor GL
*Os01g0127450*	*-*	2.42	–	–	Similar to MYBL2 (ARABIDOPSIS MYB-LIKE 2); DNA binding/transcription factor
*Os01g0191900*	R2R3-MYB	2.28	–	–	Similar to Blind
*Os03g0410000*	*OsMyb3*	2.06	–	–	Conserved hypothetical protein
*Os01g0709000*	*-*	–	-4.46		Similar to Transcription factor MYB1
*Os01g0975300*	*OsMYB48*	–	-1.68		MYB-related transcription factor, Drought and salinity tolerance
*Os12g0175400*	*R2R3-MYB*	–	-2.49	–	Similar to *OsMYB2*
*Os04g0557200*	*OsbHLH015*	6.22	–	6.51	Similar to Anthocyanin regulatory B protein (Fragment)
*Os12g0599550*	*OsbHLH116*	5.15	–	4.59	Similar to Helix-loop-helix DNA-binding domain containing protein
*Os04g0429300*	*OsbHLH145*	–	-1.64	-1.31	Similar to OSIGBa0093L02.4 protein
*Os01g0383700*	*OsWD40-14*	2.93	2.94	–	Similar to LEC14B protein
*Os02g0663300*	*OsWD40-48*	3.56	3.12	–	BEACH domain domain containing protein
*Os02g0682500*	*OsWD40-50*	2.15	–	-1.76	Similar to TRANSPARENT TESTA GLABRA 1 protein (TTG1 protein)
*Os06g0644600*	*OsWD40-131*	1.66	1.86	–	Similar to predicted protein
*Os11g0212900*	*OsWD40-187*	7.52	7.68	–	Serine/threonine protein kinase-related domain containing protein
*Os01g0607600*	*OsWD40-18*	–	-1.73	-1.45	WD40 repeat domain containing protein
*Os01g0177100*	*OsWD40-4*	4.12	–	–	Similar to STYLOSA protein

#### 3.6.3 Identification of major transporter genes for anthocyanin storage process

**Figure 11:**
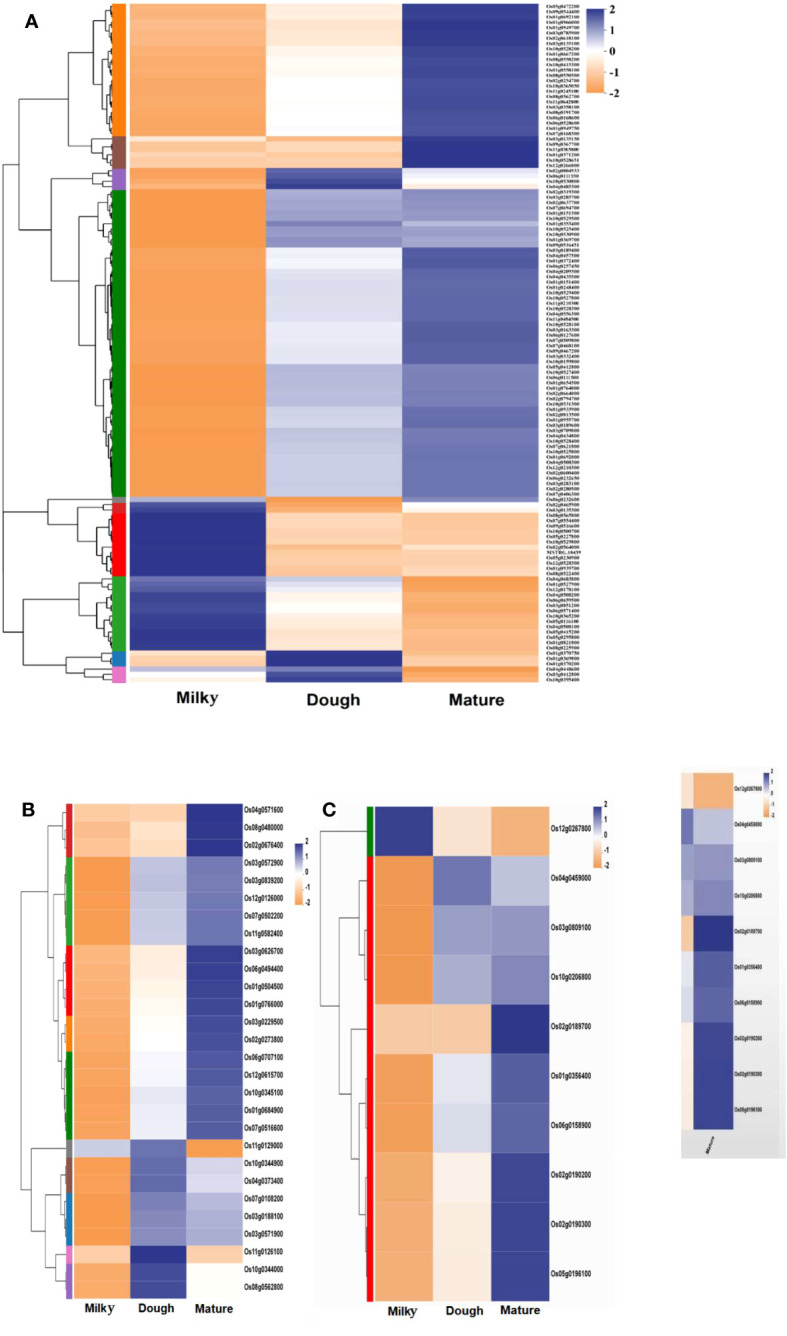
Heatmap of significantly expressed transporter gene family; **(A)** heatmap of significantly regulated glutathione transporter gene family; **(B)** heatmap of significantly regulated MATE transporter gene family; **(C)** heatmap of significantly regulated MRP transporter gene family. Each column in the figure represents a sample, each row represents a gene, and the color in the figure indicates the expression value of the gene after standardization in each sample; red represents the gene in the sample with a higher expression, blue represents a low expression, and for the specific expression size change trend, please see the number under the upper left color bar. On the left is the dendrogram of gene clustering and the module diagram of sub-clustering; on the right is the name of the gene; the closer the two gene branches are, the closer they are expressed; the upper part is the dendrogram of the sample cluster; the lower part is the name of the sample; the closer the two sample branches are, the closer the expression patterns of all genes in the two samples are, that is, the closer the gene expression trend is.

Anthocyanins are synthesized in the cytoplasm and stored in the vacuole. Three main transporter families were investigated: the glutathione family (GST), multi-drug, multi-antimicrobial extrusion (MATE), and multi-drug-resistant protein (MRP), an ABC transporter family. A total of 134 GSTs, 32 MATEs, and 11 MRP transporters that were significantly regulated were identified. Among the GST group, only 17 DEGs were shared in all groups (milky vs. dough, dough vs. mature, and milky vs. mature); 60 DEGs were shared between milky vs. dough and milky vs. mature; 6 DEGs were shared between milky vs. mature and dough vs. mature; and others were found only in one comparison group (**Supplemental Table 8**). Among all comparisons for MATEs, six DEGs were shared in all groups (milky vs. dough, dough vs. mature, and milky vs. mature); nine DEGs were shared between milky vs. dough and milky vs. mature; one DEG was shared between milky vs. dough and dough vs. mature; and other DEGs were found only in one of the comparison groups (**Supplemental Table 9**). The expression profiling of these DEGs varied throughout the developmental stages. For the MRP family, three DEGs were shared across all comparison groups (milky vs. dough, dough vs. mature, and milky vs. mature); four DEGs were shared between milky vs. dough and milky vs. mature; one DEG was shared between milky vs. dough and dough vs. mature; and others were found only in one comparison group (**Supplemental Table 10**). Based on the above analysis, most of the DEGs’ expression did not follow the anthocyanin biosynthesis trends. Nevertheless, some genes were highly upregulated in the dough stage, consistent with the amount of anthocyanin. The heatmap profiling revealed 10 GSTs (*Os02g0804933, Os01g0353400, Os01g0369800, Os10g0530800, Os06g0111550, Os01g0370200, Os01g0370750, Os03g0412800, Os04g0485300, Os10g0395400*), six MATEs *(Os10g0344000, Os10g0344900, Os04g0373400, Os08g0562800, Os11g0126100, Os11g0129000*), and one MRP (*Os04g0459000*) highly up-regulated at the dough stage and down-regulated in other stages (**Figure 11**). This result suggests that these genes may greatly contribute to the anthocyanin biosynthesis pathway.

### 3.7 Validation of transcriptome outcome and gene expression patterns

**Figure 12:**
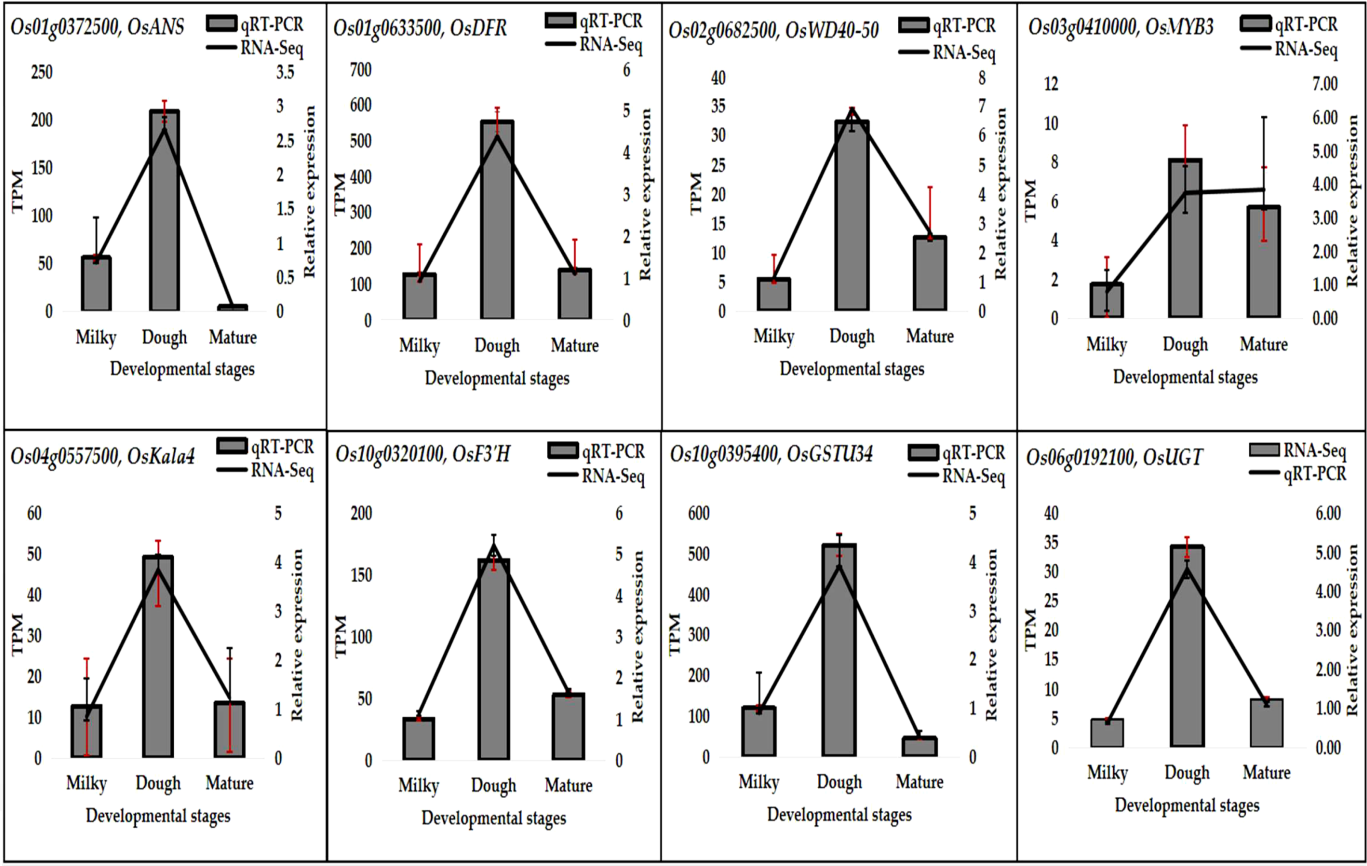
qRT-PCR results of eight DEGs at developmental stages.

Eight important genes were selected, and their expressions were analyzed through qRT-PCR. The results indicated that the gene expression pattern was similar to the RNA-Seq result, although the quantitative difference was not the same. The expression of all these genes was at its peak at the dough stage, consistent with anthocyanin accumulation (**Figure 12**). This result suggests that anthocyanins greatly correlate with gene expression.

## 4 Discussion

Black rice is a rich, functional food with potent antioxidant properties. Besides their health properties, anthocyanins found in black rice and other pigmented crops (fruits and vegetables) have great importance in the food industry. They are often used as alternatives for food colorants (Turturica et al., 2015) and pH indicators, which change color from red to blue in acidic and alkaline environments, respectively. A study reported that the change in the caryopsis coloration during black rice development is due to the accumulation of anthocyanin (Jiamyangyuen et al., 2017). In a recent study, transcriptome and metabolite analysis allowed for the understanding of fruit coloration mechanism and identified 10 structural genes related to anthocyanin biosynthesis in *Schisandra chinensis* (Li et al., 2022). Since several genes regulate anthocyanin biosynthesis in rice, keeping track of changes in anthocyanin content is essential to comprehending the mechanisms underlying anthocyanin biosynthesis and identifying the essential genes.

### 4.1 Anthocyanin accumulation relatedness with structural gene expression

This study showed that the caryopsis coloration started to change about 8 DAF. This suggest that the anthocyanin accumulation is initiated at this period, which may coincide with the activation of some important genes involved in the biosynthesis pathway. The amount of anthocyanin reached its peak 10 days after starting and then decreased significantly so that at the fully ripe stage, which is the common harvesting time (35 DAF), only 20% of total anthocyanin remained. Although the amount of C3G was significantly higher than that of P3G, the accumulation curve was similar for both types, indicating that the two main anthocyanins were linked. This result was similar to previous studies reporting that The amount of anthocyanins increased during the flower development stages and then decreased in the senescence stage (Wu et al., 2019; Park et al., 2021).

Advanced plant genomic research has reported RNA sequencing as a high-throughput method to predict new genes, gene function, and some important genes involved in a specific pathway. For example, transcriptome analysis showed the role of anthocyanin in plant coloring and some key genes in anthocyanin biosynthesis in grapes (Li et al., 2018), red leaves of poplars (Chen et al., 2021), red pears (Wu et al., 2019), and Lycoris radiata (Wang et al., 2021). Chen and co-authors, (2022) used data from the transcriptome to show that the expression levels of F3H, DFR, ANS, F3′H, and the transcription factor C1 were strongly linked to the amount of anthocyanin in young diploid maize embryos(Chen et al., 2022)

In the present study, we performed transcriptome analysis at different stages of black rice caryopsis development to identify key genes for anthocyanin at each phase. The result showed that as caryopsis developed, the total number of DEGs increased. We identified 18087, 16886, and 14408 DEGs in the mature, dough, and milky stages, respectively. The number of up-regulated genes was significantly higher than the number of down-regulated genes. Several important processes involving the recruitment of an increasing number of genes occurred during caryopsis formation and maturation, according to our findings. The KEGG enrichment revealed that the majority of the DEGs were involved in the metabolism of sugar, lipids, amino acids, and transport. This is consistent with physiological activities in rice during grain formation, and these metabolites are very important for caryopsis development. Besides, the results revealed that the phenylalanine metabolism pathway (map00360), glutathione metabolism pathway (map00480), and flavonoid metabolism pathway (map00941) were enriched (**Supplemental Table 11**). In plants, phenylalanine is vital in the formation of the secondary metabolites competing in anthocyanins, proanthocyanins, flavonols, and isoflavonol synthesis because they all have a similar metabolism pathway called the phenylpropanoid pathway (Ferrer et al., 2008). Since anthocyanin is well-known to modulate color formation in black rice and the variation in coloration was revealed to emanate mainly from gene expressions and pigment storage mechanisms (Grotewold, 2006; Cui et al., 2021), we focused on the anthocyanin biosynthesis pathway of the flavonoid biosynthesis pathway. Furthermore, we pointed out the anthocyanin-related structural genes *PAL, C4L, C4H, CHS, CHI, FLS, F3H, F3’H, DFR, LDOX, ANR, UGT, UFGT, ROMT*, and *3GT* that were associated with anthocyanin. Our result was consistent with Pu and co-workers showing that the expression pattern of structural genes (such as the *ANS, DFR, CHS, F3H, PAL4, OMT1*, and *4CL4*) and key transcription factors (such as the TT8 and WRKYs) through RNA-seq and qRT-PCR analysis, was higher in purple radish leaves compared to green (Pu et al., 2022).

**Figure 13:**
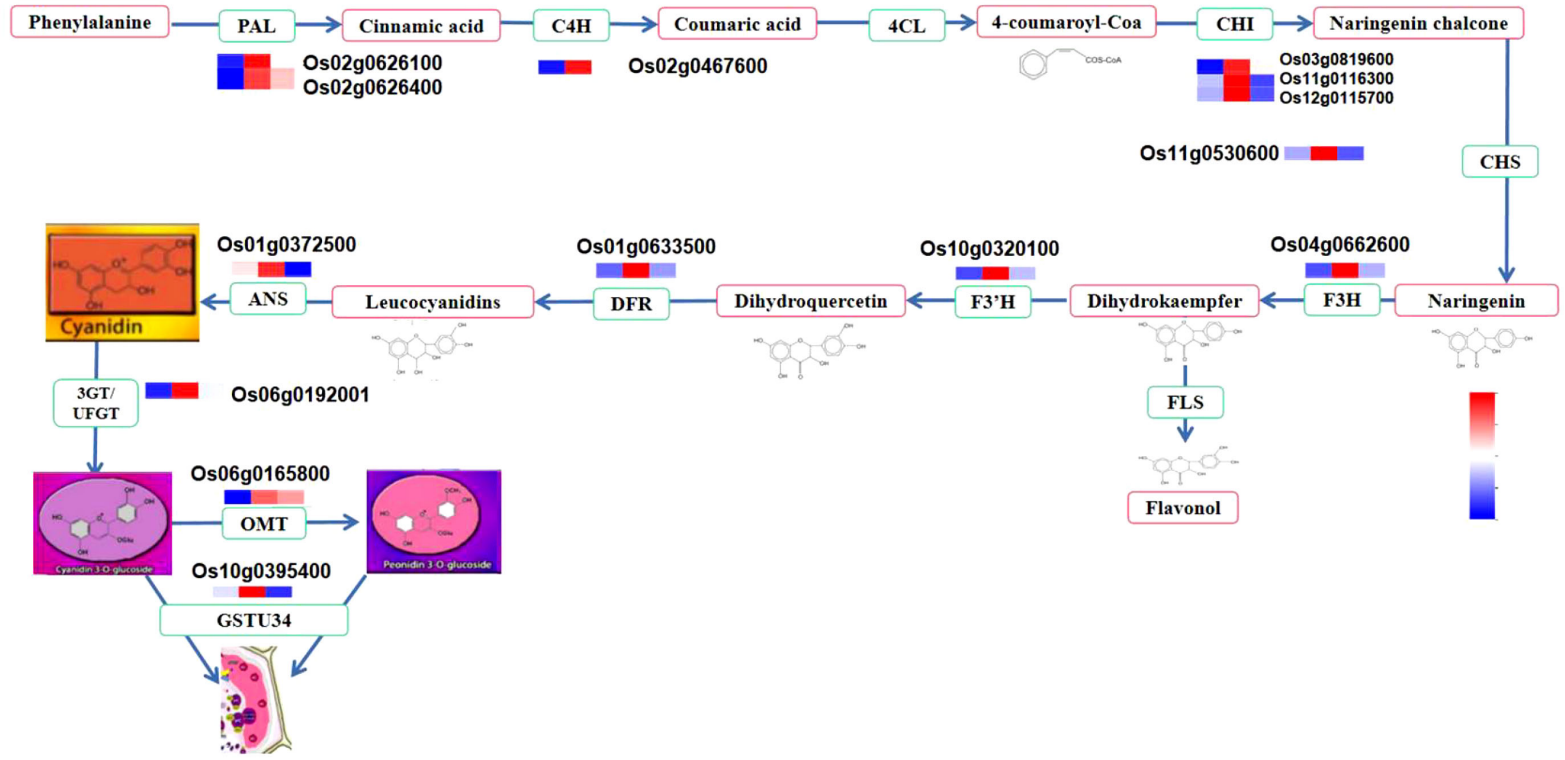
Anthocyanin metabolism pathway and structural gene expression at different stages. The fold change of gene expression was normalized by log2 transform and scaled to the same range to represent color scores.

Some genes may have several copies, as we identified different genes with the same annotation and coding for the same or similar products. For example, three genes were annotated as *PAL*, all coding for phenylalanine ammonia-lyase; two genes were annotated as *CHI*, coding for chalcone isomerase; etc. (**Table 3**). Although all these genes contributed to anthocyanin synthesis and were positively correlated with anthocyanin content, *PAL (Os02g0626100, Os02g0626400), ANS (Os01g0372500), CHS (Os11g0530600), DFR (Os01g0633500), F3H-1 (Os04g0662600)*, and *F3’H (Os10g0320100)* were identified as the most significant functional genes with a great contribution to the increasing amount of anthocyanin from milky to dough phase, which showed high content at dough stage synthesis. This showed that the simultaneous expression of these genes boosted the production of anthocyanin (**Figure 13**) and was linked to the change in the color of the caryopsis, which gets darker as it matures. Upstream genes were found to be *PAL, CHS, CHI*, and *F3’H* were identified as upstream genes which are also involved in the synthesis of other flavonoids. Downstream genes, *DFR* and *ANS*, enter anthocyanin biosynthesis directly and are involved in the synthesis of anthocyanin precursors (Chen et al., 2019). This suggests that these genes were the main contributors and are important at this stage. This was consistent with the previous research showing that overexpression of *LrDFR1* increased anthocyanin accumulation in lycoris petals and tobacco leaves (Wang et al., 2021), and CRISPR/Cas9-targeted mutagenesis of *F3′H* and *DFR* resulted in a change in seed coloration and anthocyanin content in black rice (Jung et al., 2019). *F3’H* is involved in the synthesis of dihydroquercetin; *DFR* catalyzes the reduction of dihydroquercetin to leucoanthocyanidins, and *ANS* catalyzes the synthesis of anthocyanidins, the immediate precursor of anthocyanin. Our investigation suggested that the increases and decreases in the amount of anthocyanin were mainly due to the expression of genes. After the transition from the mature to fully ripe phase, the amount of anthocyanin greatly decreased, suggesting that anthocyanin may be unstable and easily degraded when the caryopsis is getting mature and after the peak concentration is reached. Thus, in order to achieve high amounts of anthocyanin for different uses, rice should be harvested before 20 DAF, just after the peak accumulation is reached.

### 4.2 Anthocyanin regulation during caryopsis development

Transcription factors are very important in regulating the activity of structural genes during anthocyanin accumulation. Different classes of TFs were predicted in this study, including MYB, ERF, NAC, WRKY, B3, M-type, HB-other, GRAS, bHLH, and FAR1, with MYB as the most abundant (**Figure 10**). However, we mainly focused on the MYB and bHLH families and the WD-repeat families since it is well-known that anthocyanin is regulated at different levels by the complex MYB-bHLH-WD40. In this complex, MYB is a highly conserved signature motif and was reported as one of the main components of the complex, binding the DNA (Zhao et al., 2015; Kavas et al., 2022; Yang et al., 2023). In this study, we identified *Os03g0410000* (*OsMyb3*) as the main MYB TF involved in caryopsis pigmentation. The expression was significantly upregulated from the milky to the dough stage, which is the active phase for anthocyanin accumulation, while it was downregulated from the dough to the mature stage. In addition, *OsMyb3* interacted with nine genes, including transcription factors *OsbHLH* and *OsWD40* and structural genes (*OsANS, OsDFR, OsUFGT, OsF3H, OsCHI*), and a P450. A previous study identified *OsMyb3* as *Kala3*, which interacted with *Kala4* in the anthocyanin regulatory complex in rice pericarp (Oikawa et al., 2015). Our result is in line with this research, suggesting that *OsMyb3* is a key gene that positively modulates the expression of anthocyanin synthesis.

**Figure 14:**
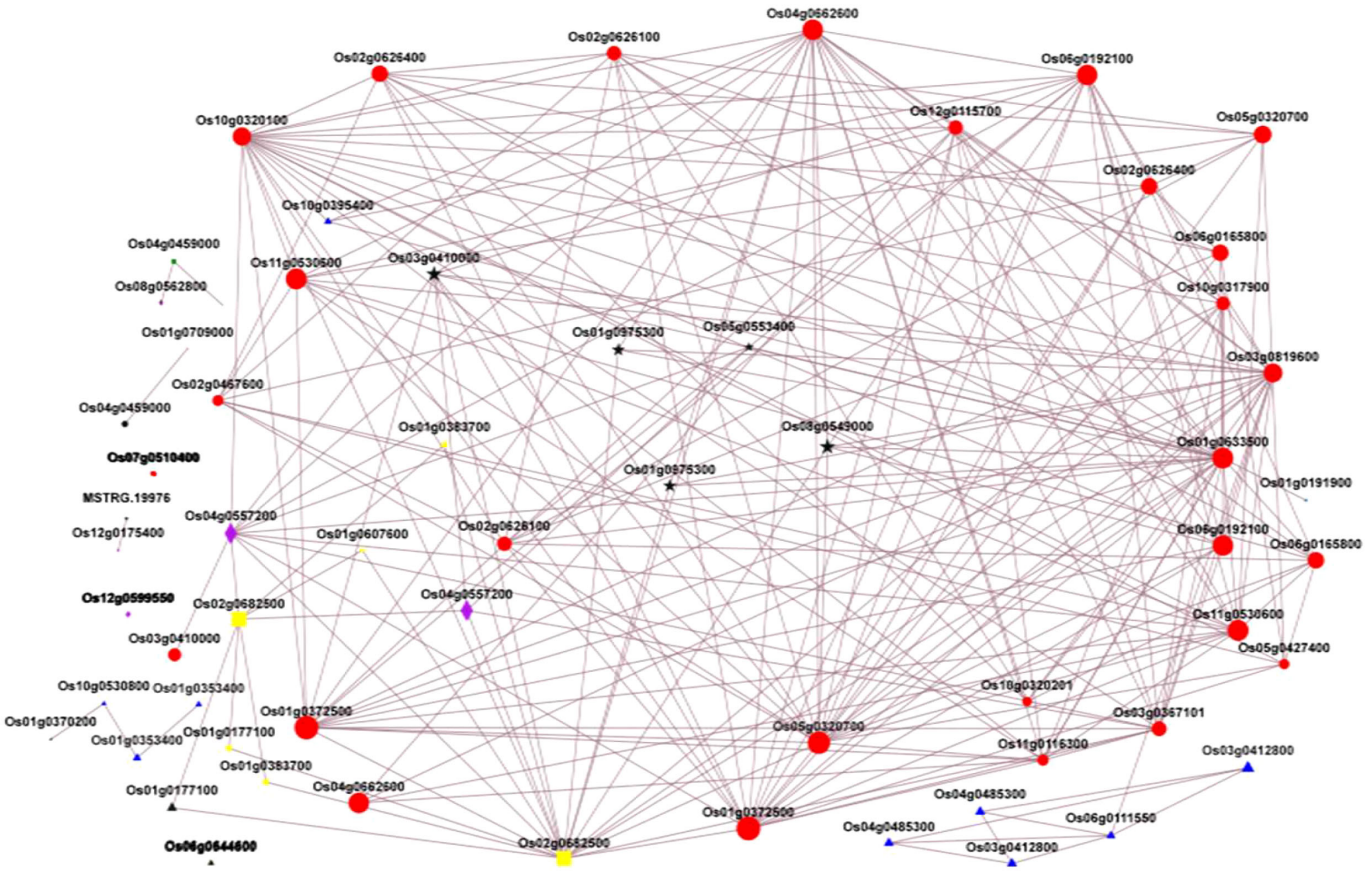
Protein interaction network diagram; Nodes represent genes, illustrated with different color, which represents gene class, and edges indicate interactions between two genes. Red nodes, structural genes; yellow nodes, WD40 TFs; Black nodes, MYB TFs; blue nodes, GST transporters; green nodes, ABC transporters; purple nodes, bHLH TFs; and orange nodes; MATE TFs. The size of a node is proportional to the connectivity (i.e., degree) of that node, i.e., the more edges connected to this node, the larger the degree and the larger the node, indicating the greater the importance of genes in the network.

The bHLH is important to activate the MBW complex. In this study, we found Os04g0557200 (OsbHLH015), a bHLH TF that was similar to the anthocyanin regulatory B protein fragment. Previous research revealed a locus named *Kala4* harboring three alleles: *Os04g0557200, Os04g0557500*, and *Os04g0557800* (Oikawa et al., 2015). So far, *Os04g0557500* has been renamed *Kala4* because it was reported as the main gene in this locus. In many studies, *Kala4/OsB2* was called *Os04g0557500, OsB1*, an important gene for purple leaves, was called *Os04g0557800* (Oikawa et al., 2015; Sun et al., 2018; Xia et al., 2021), and *Os04g0557200* (*OsbHLH015*) was never mentioned. This study highlighted that *Os04g0557200* could be associated with caryopsis coloration. The expression profiling showed that *OsbHLH015* was up-regulated during the milky and dough stages but not at the mature stage, which is consistent with the anthocyanin accumulation. The expression was 74 times higher at the dough stage, when the anthocyanin reached its peak, compared with the milky stage, and decreased at the mature stage. The protein interaction showed that *OsbHLH015* may interact with structural genes (*OsANS, OsF3,5H, OsF3H, OsUFGT*, and *OsDFR*) and transcription factors such as MYB (*Os03g0410000, Os011g0530600, Os05g0553400*), and WD40 (*Os02g0682500*) (**Figure 14**). Our result was consistent with previous research, which reported that Kala4 interacted with MYB, WD40, and structural genes (Maeda et al., 2014; Oikawa et al., 2015; Sun et al., 2018; Xia et al., 2021; Yang et al., 2021).

In a recent study, WD-40 was reported as the scalfolding protein that interacts with bHLH (Mackon et al., 2021a; Yang et al., 2021). Based on expression levels at different stages from transcriptome data, seven WD40 TFs were predicted, with *OsWD40-50* (*Os02g0682500*) being the most representative. The *OsWD40-50* was similar to the transparent testa glabra 1 (TTG1). According to Sun and co-workers. (2018), *OsTTG1* was revealed not to be a necessary gene for anthocyanin biosynthesis (Sun et al., 2018). However, in our study, the interaction analysis showed that this gene had the highest interaction with 15 other proteins, including *OsMyb3*, *OsbHLH05*, and several structural genes (**Figure 14**). The fold-change analysis revealed that *OsWD40-50* was expressed at all stages (milk, dough, and mature), but the highest expression was recorded at the dough stage, which is consistent with anthocyanin accumulation. This suggests that this gene may be highly associated with anthocyanin biosynthesis at all developmental stages. Our findings were consistent with those of Yang et al. (2021), who discovered that *OsWD40-50* was a critical regulator of anthocyanin biosynthesis and that when this gene was knocked out, rice grains almost turned colorless (Yang et al., 2021).

### 4.3 GST is the main anthocyanin carrier in rice caryopsis

Anthocyanin is synthesized at the external surface of the endoplasmic reticulum and stored inside the vacuole. The trafficking and storage mechanisms of anthocyanin inside the vacuole lumen are similar to xenobiotic transport (Coleman et al., 1997). This mechanism ensures that cells get rid of toxins and prevents oxidation (Marrs et al., 1995), which can be harmful to the cells, hindering the efficiency of their production (Roytrakul and Verpoorte, 2007), and enhances the anthocyanin’s ability to function as pigments when present in the acidic vacuolar environment (Verweij et al., 2008). The mechanism of anthocyanin accumulation inside the vacuole involved several transporters. In the present study, we investigated three main transporter families, including GST, MATE, and the ABC transporter family. Among those transporters, the GST and MATE classes showed obvious differences with others at the three developmental stages, which were consistent with the amount of anthocyanin (**Figure 9**). The fold change analysis at different stages showed that *Os10g0395400*, also known as *OsGSTU34* (thau group) with a thioredoxin fold domain, and *Os08g0562800*, which is similar to transparent testa protein 12, are involved in the biosynthesis of anthocyanin (**Supplemental Table 12**). In previous research, *Os08g0562800* was annotated as *OsMATE34* (Huang et al., 2019), a putative anthocyanin transporter (Mackon et al., 2021c). However, its function is still not clear. In general, the MATE family is involved in cells’ detoxification through metabolites, alkaloid sequestration, hormones, and organic acid transport. The protein interaction network showed that *Os08g0562800* interacted with one gene, *MDR13*, which was called a multidrug-resistance (MDR)-like ATP Binding Cassette B (ABCB) transporter, P-glycoprotein, auxin transporter, and iron homeostasis. Still, some studies showed that MATE was involved in anthocyanin in *Vitis vinifera* (Gomez et al., 2009), *Medicago truncatula* (Zhao et al., 2011), and *Raphanus sativus* (M’mbone et al., 2018), suggesting that *Os08g0562800* may also be associated with anthocyanin in rice. Compared with *Os08g0562800*, *Os10g0395400 (OsGSTU34*) interacted with four genes: *Os10g0320100* (flavonoid-3’-hydroxylase), *Os01g0633500* (dihydroflavonol-4-reductase), Os06g0192100 (uridine flavonoid glycosyl-transferase), and *Os04g0662600* (flavonoid-3’-hydroxylase). These four genes are important structural genes, named late biosynthesis genes because they are involved in the reaction that directly leads to anthocyanin formation and are thus determinants for anthocyanin biosynthesis (Oh et al., 2018; Yang et al., 2019; Mackon et al., 2021c; Xia et al., 2021). The expression profiles of these genes correlated with the amount of anthocyanin (**Supplemental Table 12**). GSTs are multifunctional enzymes that participate in the accumulation of secondary metabolites *via* glutathione conjugation to various electrophilic compounds (Dixon et al., 2013). Several studies reported the GST family as an important anthocyanin transporter in apples (Jiang et al., 2019), petunia (Larsen et al., 2003), A. thaliana (Mueller et al., 2000), and maize (Marrs et al., 1995). In this study, the expression of *OsGSTU34* was highest at the dough stage and drastically decreased at the mature stage. This indicated that the transport activity was important when the peak was obtained, suggesting that *OsGSTU34* was the most important anthocyanin, which plays a vital role in anthocyanin accumulation in rice caryopsis.

## 5 Conclusion

Our investigation revealed that anthocyanin accumulation in rice caryopsis mostly happens during the first 20 days after flowering (7–20 DAF). This accumulation was greatly associated with anthocyanin biosynthesis gene expression, whose peak was attained at the dough stage 18 days after flowering. Our results suggested that to achieve rice production with high anthocyanin content, the caryopsis could be harvested before 20 DAF. However, further study should be carried out to assess the stability and quality of anthocyanin extract from the dough stage (20 DAF) to the fully ripe stage (35 DAF) in order to align high quantity with good quality. The darker color of caryopsis observed from the mature to fully ripe stage does not necessarily reflect the quantity of anthocyanin. So, it is possible that this color may rather reflect the degradation rate of anthocyanin, which is completely bound to grain particles and cannot be released in the extract. This study showed that the combination of metabolite profiling and transcriptome analysis could be a useful way to decipher the key genes involved in phytochemical synthesis. The present findings provide valuable information for anthocyanin metabolism and potential candidate genes for further genetic manipulation of anthocyanin biosynthesis in rice caryopsis.

## Data availability statement

The datasets presented in this study can be found in online repositories. The names of the repository/repositories and accession number(s) can be found in https://www.ncbi.nlm.nih.gov/sra, PRJNA967198. The original contributions presented in the study are included in the article/**Supplementary Material**. Further inquiries can be directed to the corresponding author.

## Author contributions

Conceptualization: EM and PL. Data curation: EM, GCJDEM, YY, and YG. Formal analysis: EM, YM, and GCJDEM. Funding acquisition: PL. Investigation: EM, GCJDEM, YY, YM, YG, XD and TH. Methodology: EM, PL. Resources: PL. Software: EM, YM, and YY. Supervision: PL. Validation: EM, and PL. Writing—original draft: EM. Writing—review and editing: EM, GCJDEM, YY, YM, YG, XD, THJ and PL. All authors have read and agreed to the published version of the manuscript. All authors contributed to the article and approved the submitted version.
