# Supplementary figures and images for: Integrative HPLC profiling and transcriptome analysis revealed insights into anthocyanin accumulation and key genes at three developmental stages of black rice (Oryza sativa. L) caryopsis
Source: Front Plant Sci. 2023 Aug 30;14:1211326. doi: 10.3389/fpls.2023.1211326 (PMC10505814; doi:10.3389/fpls.2023.1211326)

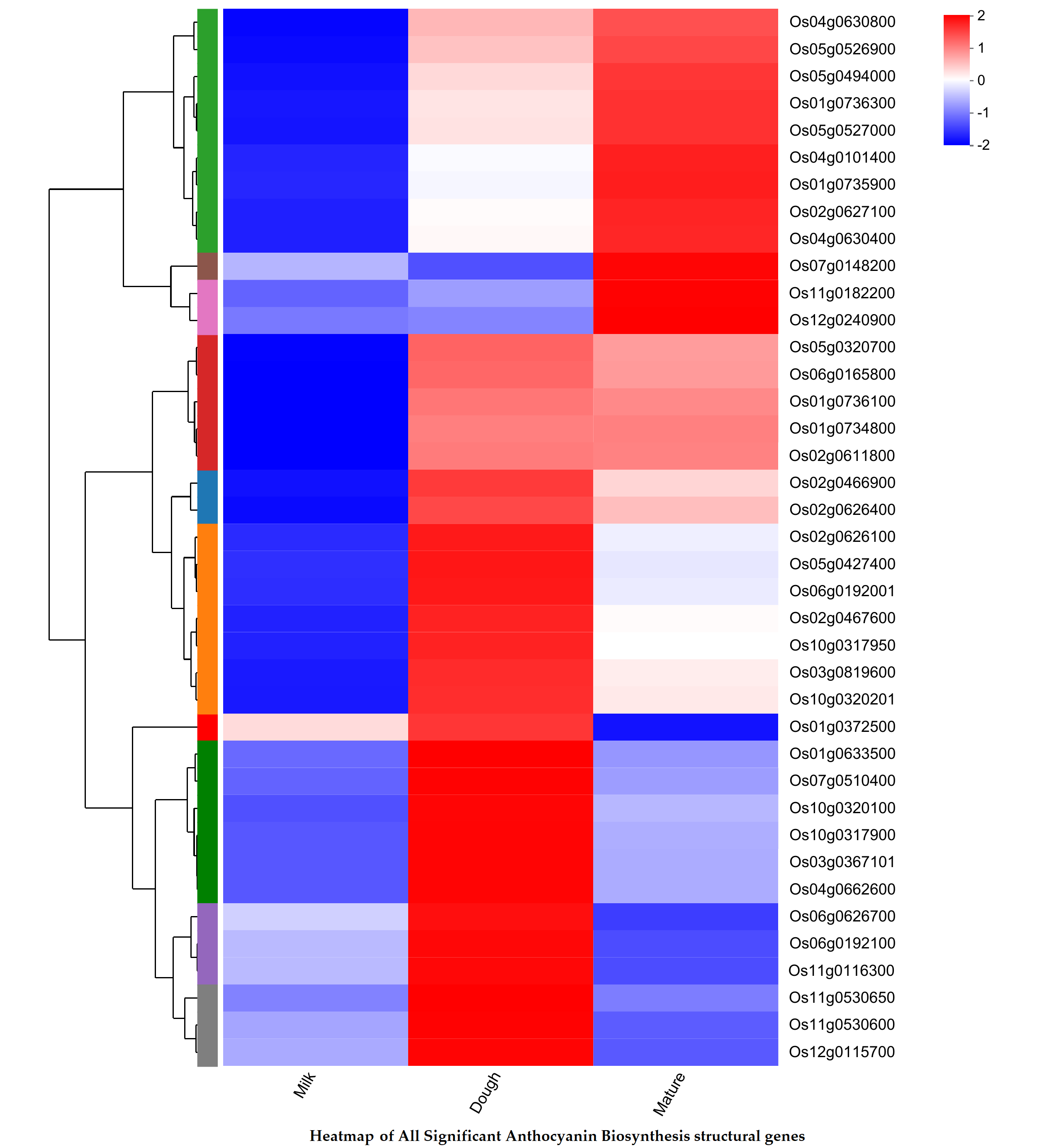

Supplement: Supplementary file 1 [file DataSheet_1.zip › supplemental fig 3. Heatmap all significant structural genes.png]

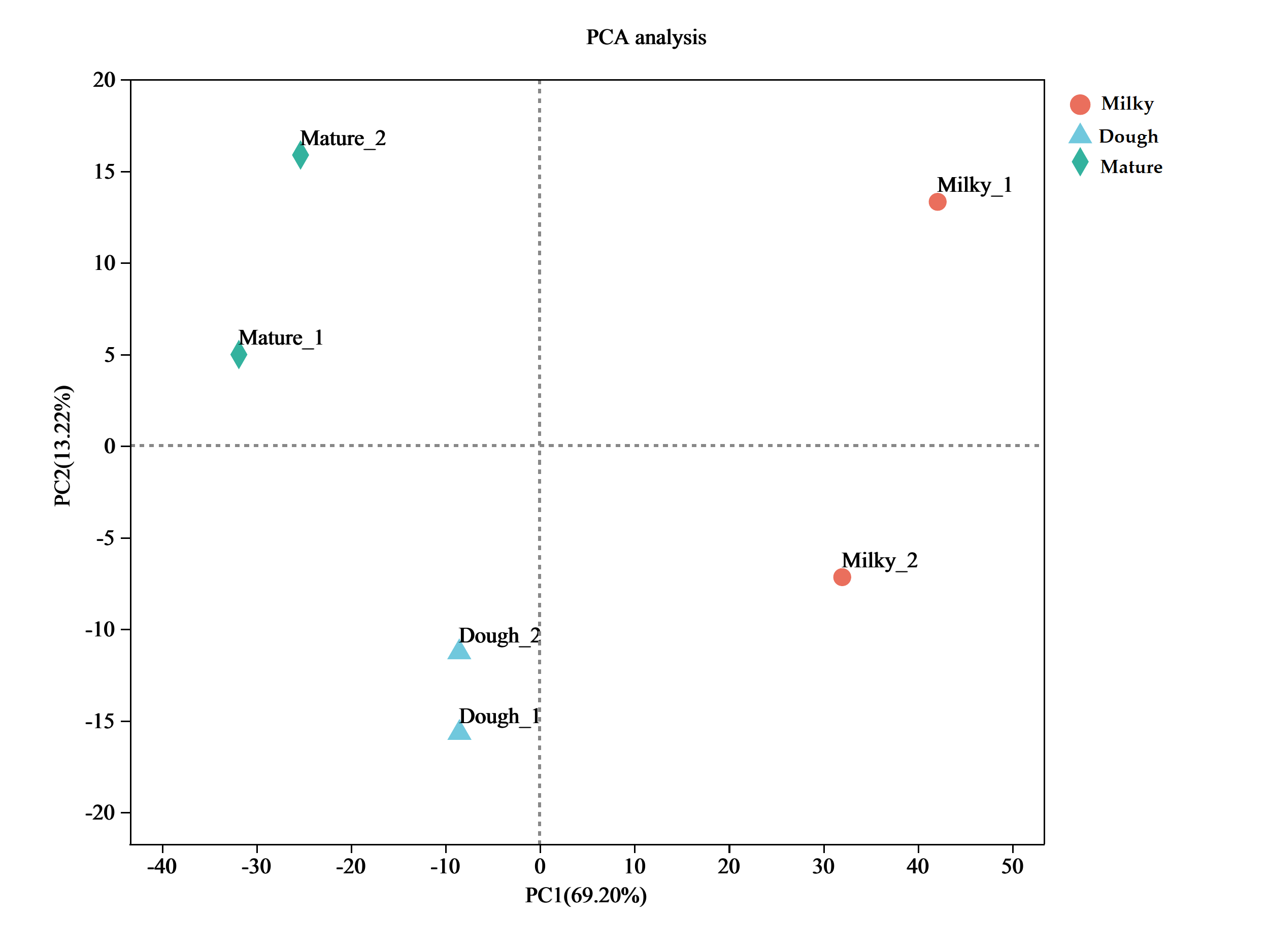

Supplement: Supplementary file 1 [file DataSheet_1.zip › Supplemental Figure 1. PCA Analysis.png]

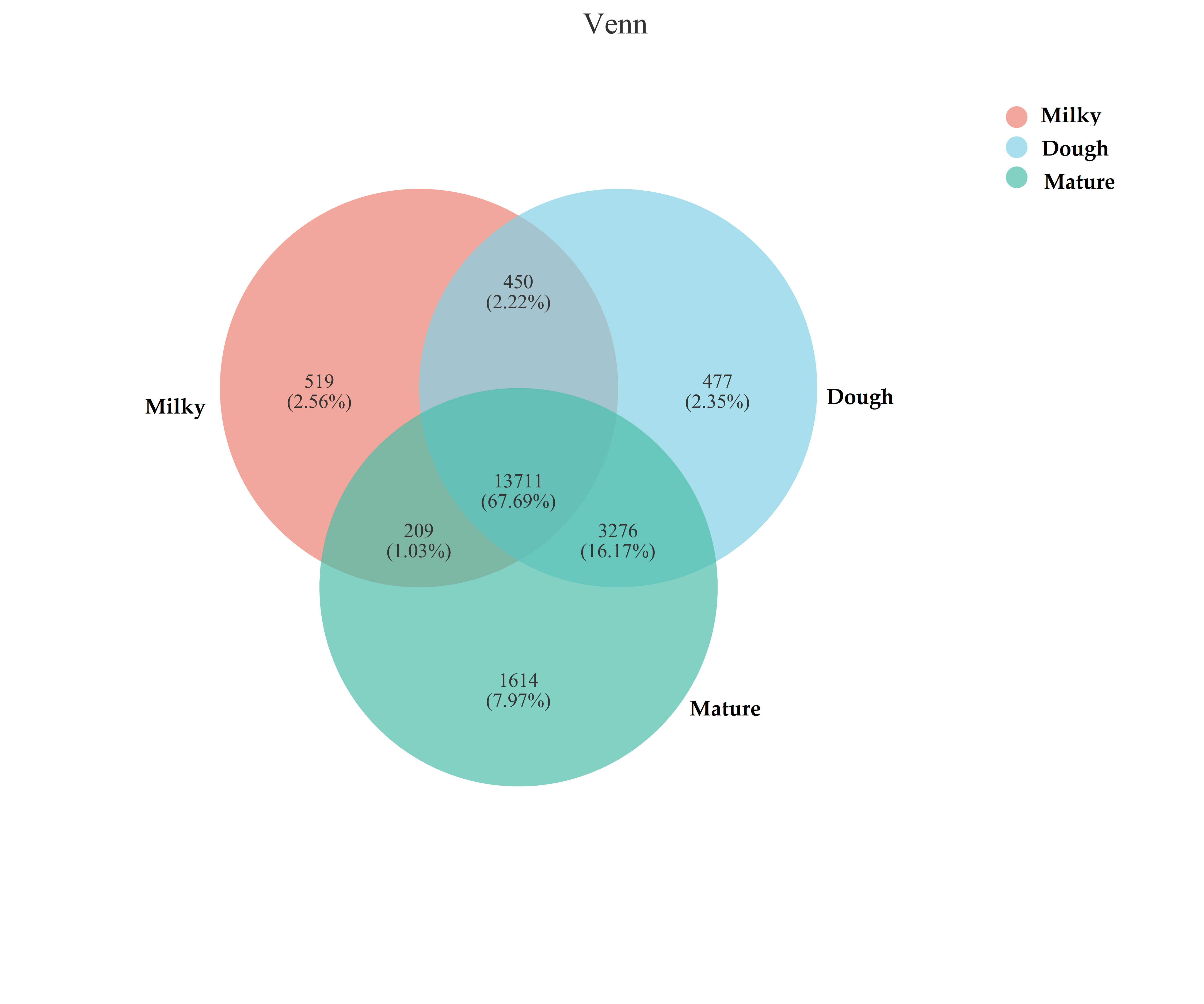

Supplement: Supplementary file 1 [file DataSheet_1.zip › supplemental figure 2. Venn Uniquely expressed genes.png]

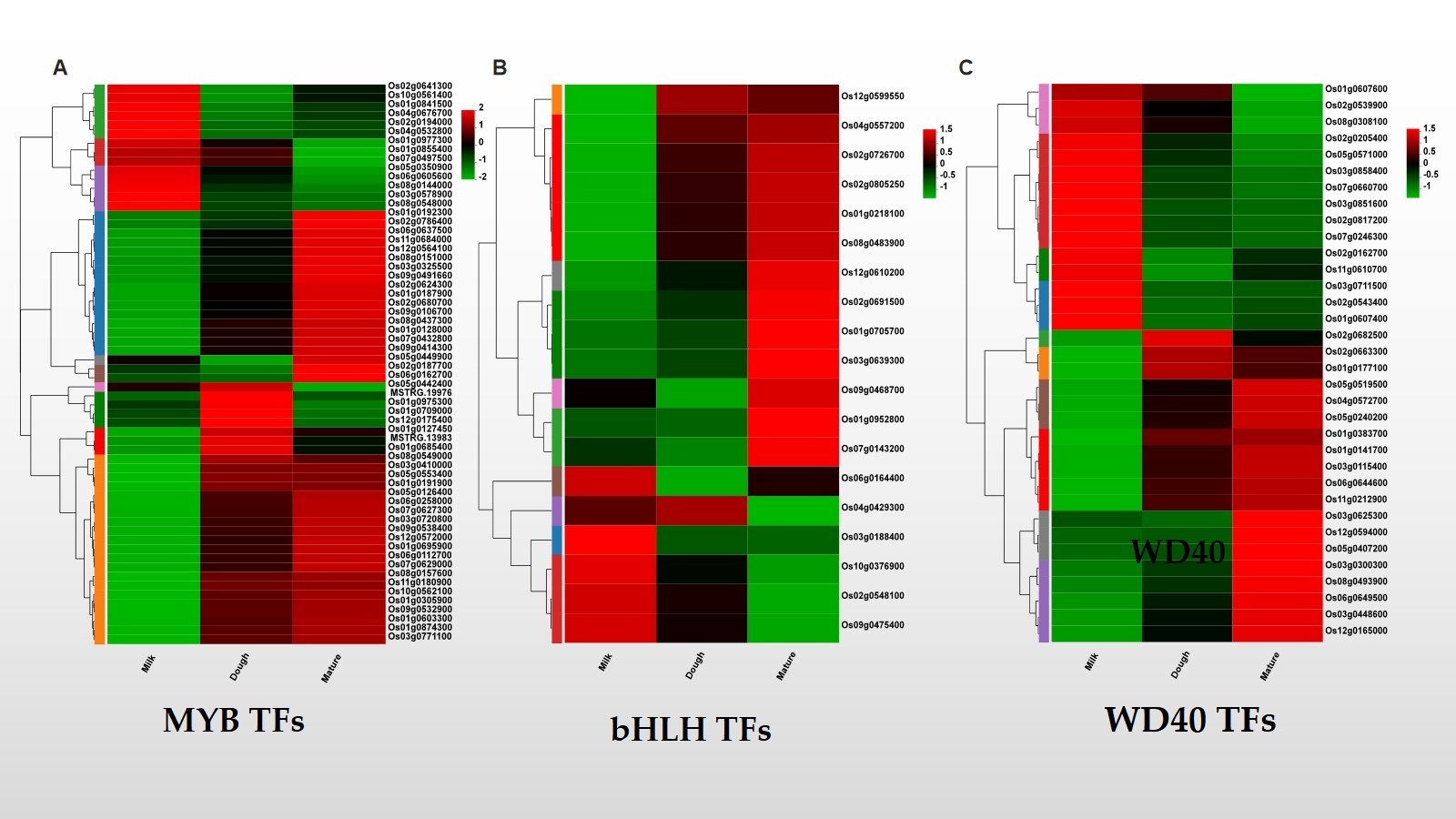

Supplement: Supplementary file 1 [file DataSheet_1.zip › supplemental figure 4. Heatmap regulatory genes.jpg]
